# Wakefulness Is Promoted during Day Time by PDFR Signalling to Dopaminergic Neurons in Drosophila melanogaster

**DOI:** 10.1523/ENEURO.0129-18.2018

**Published:** 2018-08-08

**Authors:** Sheetal Potdar, Vasu Sheeba

**Affiliations:** 1 Behavioural Neurogenetics Laboratory, Evolutionary and Organismal Biology Unit, now known as Evolutionary and Integrative Biology Unit, Jawaharlal Nehru Centre for Advanced Scientific Research, Bangalore 560064, India; 2 Behavioural Neurogenetics Laboratory, Neuroscience Unit, Jawaharlal Nehru Centre for Advanced Scientific Research, Bangalore 560064, India

**Keywords:** circadian, dopamine, PDF receptor, sleep, ventral lateral neurons, wake

## Abstract

Circadian clocks modulate timing of sleep/wake cycles in animals; however, the underlying mechanisms remain poorly understood. In *Drosophila melanogaster*, large ventral lateral neurons (l-LN_v_) are known to promote wakefulness through the action of the neuropeptide pigment dispersing factor (PDF), but the downstream targets of PDF signalling remain elusive. In a screen using downregulation or overexpression (OEX) of the gene encoding PDF receptor (*pdfr*), we found that a subset of dopaminergic neurons responds to PDF to promote wakefulness during the day. Moreover, we found that small LN_v_ (s-LN_v_) and dopaminergic neurons form synaptic contacts, and PDFR signalling inhibited dopaminergic neurons specifically during day time. We propose that these dopaminergic neurons that respond to PDFR signalling are sleep-promoting and that during the day when PDF levels are high, they are inhibited, thereby promoting wakefulness. Thus, we identify a novel circadian clock pathway that mediates wake promotion specifically during day time.

## Visual Abstract

**Figure:**
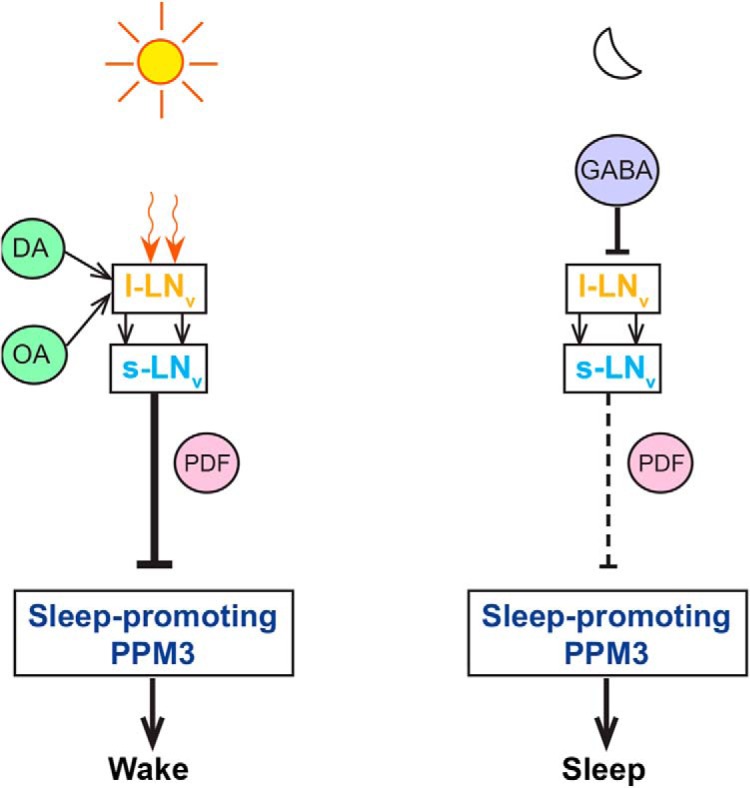

## Significance Statement

It is well established that sleep is controlled by two major factors, the circadian clock as well as a sleep homeostat. Organisms need to remain awake during appropriate times of the day to engage in activities such as feeding and mating that increase their chances of survival. How wakefulness during the day is maintained remains unknown. Here, we show that circadian clock neurons direct their inhibitory peptidergic wake-promoting signal to dopamine neurons specifically during the day time. Importantly, contact of dopamine neurons with known homeostatic structures established through previous studies underlies an important pathway bridging together circadian and homeostatic neurons in sleep/wake regulation.

## Introduction

Daily cycles in several environmental factors synchronize endogenous circadian clocks which drive rhythmic sleep/wake patterns in many organisms. Homeostatic mechanisms modulate the amount and depth of sleep, and also allow animals to recover from any sleep deprivation they may have incurred. Together, these processes control the timing and occurrence of sleep and wake states, thereby modulating sleep/wake cycles. Since the discovery that sleep behavior of *Drosophila melanogaster* is similar to mammalian sleep in several aspects (Hendricks et al., 2000; Shaw et al., 2000), many pathways and neuronal circuits involving sleep homeostat and circadian clocks have been uncovered. Genes such as *minisleep* (*mns*) and *hyperkinetic* (*hk*) encoding subunits of Shaker potassium channel function in the sleep homeostat (Cirelli et al., 2005; Bushey et al., 2007). More recently, central complex structures such as dorsal fan-shaped body (FB; Donlea et al., 2014) and the ellipsoid body (EB; Liu et al., 2016) have been shown to function as effector and modulator of the sleep homeostat, respectively. Meanwhile, mutations in core circadian clock genes such as *Clock* (*clk*) and *Cycle* (*cyc*) have been shown to cause impaired timing of sleep as they tend to become nocturnal (Kumar et al., 2012). The circadian neuropeptide pigment dispersing factor (PDF) and its receptor (PDFR) are involved in relaying wake-promoting signals from the circadian pacemaker ventral lateral neurons (LN_v_s; Parisky et al., 2008; Sheeba et al., 2008; Chung et al., 2009) in response to light input (Shang et al., 2008) as well as dopamine (Shang et al., 2011). While it has been suggested that the EB may be the downstream target of this wake-promoting PDF/PDFR signaling, the evidence in favor of the same is limited (Parisky et al., 2008).

In the recent past, in the quest to uncover output pathways of the circadian clocks that help in timing of sleep/wake cycles, a few dedicated circuits have been mapped. Most notably, timing of sleep onset at the beginning of night is a function of increased inhibition of wake-promoting large LN_v_ (l-LN_v_) by GABA (Liu et al., 2014). On the other hand, sleep is suppressed at the end of night by the action of PDF on PDFR^+^ dorsal neuron 1 (DN_1_) group of the circadian network which in turn secrete the wake-promoting neuropeptide diuretic hormone 31 (DH31; Kunst et al., 2014). Furthermore, yet another group showed that DN_1_s through glutamate modulate day-time siesta and night-time sleep by inhibiting the morning (small LN_v_; s-LN_v_) and evening (dorsal lateral neurons; LN_d_s) activity controlling circadian neurons (Guo et al., 2016). Yet, none of the studies so far have shed light on how circadian neurons may induce wakefulness during the day.

Here, we addressed this question by screening for putative downstream targets of PDFR signaling by altering the levels of *pdfr* expression in several subsets of neurons – namely, circadian neurons that are known to express *pdfr* (Hyun et al., 2005; Lear et al., 2005; Mertens et al., 2005; Im and Taghert, 2010) subsets of mushroom body (MB) neurons that are sleep- or wake-promoting (Joiner et al., 2006; Pitman et al., 2006; Cavanaugh et al., 2016), wake-promoting pars intercerebralis (PI; Foltenyi et al., 2007), sleep homeostat EB (Liu et al., 2016), and sleep-promoting FB neurons (Donlea et al., 2011) as well as aminergic neuronal groups, most of which are reported to be wake-promoting (Kume et al., 2005; Crocker et al., 2010). Strikingly, we found that a subset of dopaminergic neurons responds to changes in *pdfr* expression by changing the levels of day-time sleep, increasing *pdfr* levels decreases day-time sleep and vice versa. Moreover, we find that PDF^+^ and dopaminergic neurons form synaptic contacts with one another, along with the possibility of the former inhibiting the latter. Thus, our results uncover a dedicated pathway involving signaling from the PDF^+^ neurons perhaps to the PPM3 dopaminergic neurons in the regulation of wakefulness during the day.

## Materials and Methods

### Fly strains

Fly strains used along with their source information is listed in Table 1. Briefly, flies were maintained on standard cornmeal medium under LD12:12 at 25°C. All flies used for sleep measurements have been back-crossed to the standard *Iso*31 or *w*^1118^ (BDSC #5905) background for at least five generations. *Pdfr*^5304^, *Pdfr*^3369^, *UAS Pdfr RNAi*, *UAS dicer*, *UAS Pdfr*, and *TH GAL4* have been back-crossed to *w*^1118^ for seven generations.

**Table 1.** Details of fly stocks used in the study.

Fly strains	Source	RRID
*w* ^1118^	BDSC #5905	BDSC_5905
*Pdfr* ^5304^	BDSC #33068	BDSC_33068
*Pdfr* ^3369^	BDSC #33069	BDSC_33069
*Pdfr (B) GAL4*	Paul Taghert	BDSC_68215
*Cry-39 GAL4*	Todd Holmes	N/A
*Dvpdf GAL4*	Michael Rosbash	N/A
*Pdf GAL4*	Todd Holmes	BDSC_6900
*Clk 9M GAL4*	Fumika Hamada	BDSC_41810
*Clk 4.1M GAL4*	BDSC #36316	BDSC_36316
*Clk 4.5F GAL4*	BDSC #37526	BDSC_37526
*OK107 GAL4*	NCBS	N/A
*201y GAL4*	BDSC #4440, NCBS	BDSC_4440
*c309 GAL4*	BDSC #6906, NCBS	BDSC_6906
*c747 GAL4*	BDSC #6494, NCBS	BDSC_6494
*30y GAL4*	BDSC #30818	BDSC_30818
*Dilp2 GAL4*	Amita Sehgal	N/A
*Kurs45 GAL4*	Gunter Korge	N/A
*Kurs58 GAL4*	Gunter Korge	N/A
*Mai281 GAL4*	Gunter Korge	N/A
*Mai301 GAL4*	Gunter Korge	N/A
*121y GAL4*	BDSC #30815	BDSC_30815
*104y GAL4*	NCBS	N/A
*c5 GAL4*	BDSC #30839	BDSC_30839
*c119 GAL4*	BDSC #30824	BDSC_30824
*c232 GAL4*	BDSC #30828	BDSC_30828
*Ddc GAL4*	BDSC #7009	BDSC_7009
*TH GAL4*	BDSC #8848	BDSC_8848
*Tdc2 GAL4*	BDSC #9313, NCBS	BDSC_9313
*Npf GAL4*	Charlotte Helfrich-Forster	BDSC_25682
*UAS Pdfr RNAi*	VDRC, KK/110677	N/A
*UAS dicer*	BDSC #24651	BDSC_24651
*UAS Pdfr*	Paul Taghert	N/A
*TH-A GAL4*	Gaiti Hasan and Mark Wu	N/A
*TH-C' GAL4*	Gaiti Hasan and Mark Wu	N/A
*TH-C1 GAL4*	Gaiti Hasan and Mark Wu	N/A
*TH-D' GAL4*	Gaiti Hasan and Mark Wu	N/A
*TH-D1 GAL4*	Gaiti Hasan and Mark Wu	N/A
*TH-D4 GAL4*	Gaiti Hasan and Mark Wu	N/A
*TH-F2 GAL4*	Gaiti Hasan and Mark Wu	N/A
*TH-F3 GAL4*	Gaiti Hasan and Mark Wu	N/A
*TH-G1 GAL4*	Gaiti Hasan and Mark Wu	N/A
*Pdf LexA*	Michael Rosbash	N/A
*LexAop spGFP11/Cyo;UAS spGFP1-10/TM6B*	Amita Sehgal	N/A
*UAS GFP AH2*	BDSC #6874	BDSC_6874
*Pdfr Myc*	Paul Taghert	N/A
*LexAOp CD8 GFP-2A-CD8GFP;UAS mLexA VP16 NFAT,cdc1(H-2,LexAOpCD2GFP/TM6,Tb*	BDSC #66542, NCBS	BDSC_66542
*UAS NachBac*	Todd Holmes	BDSC_9467
*UAS dORKNC1*	Todd Holmes	N/A
*UAS Htt Q0A*	Todd Holmes	N/A
*UAS Htt Q128c*	Todd Holmes	N/A
*UAS reaper*	Paul Taghert	N/A
*UAS PKAR*	Daniel Kalderon	N/A
*UAS PKACA*	Daniel Kalderon	N/A
*UAS dTRPA1*	BDSC #26263, NCBS	BDSC_26263

BDSC, Bloomington *Drosophila* Stock Center, Bloomington, Indiana; NCBS, National Center for Biological Sciences, Bangalore, India; VDRC, Vienna *Drosophila* Resource Center, Vienna, Austria. *TH*-subset *GAL4*s that were generated in Mark Wu’s lab were obtained from Gaiti Hasan.

### Sleep assays

Three- to 6-d-old mated females were individually housed in glass tubes (65 mm in length, 3 mm in diameter) with sucrose medium (5% sucrose and 2% agar) on one end and cotton plug on the other and activity was recorded in DAM2 monitors (*Drosophila* activity monitoring system, Trikinetics). The DAM system works on the principle that whenever a fly crosses the middle of the tube, it breaks an infra-red beam which gets detected by infra-red sensors and recorded as activity counts. Flies were housed in light and temperature-controlled environments with 12 h of light (∼300–500 lux) and 12 h of darkness (LD12:12) at 25°C in incubators (MIR-273, Sanyo; DR-36VLC8 Percival Scientific Inc.) for a period of 3 d. Activity was binned at 1 min and sleep parameters such as day-time and night-time sleep duration, bout length and number and activity per waking minute were estimated using PySolo (Gilestro and Cirelli, 2009), while sleep profiles and sleep latency were obtained from a custom-made Microsoft Excel spreadsheet template.

### Statistical analysis

Change in day-time sleep is calculated as difference between day-time sleep of experimental flies and *GAL4* or *UAS* control flies as a percentage of day-time sleep of *GAL4* or *UAS* control flies. For comparison of sleep parameters, one-way ANOVA with genotype as fixed factor followed by *post hoc* Tukey’s HSD test was conducted. For comparison of GFP fluorescence intensity, two-way ANOVA with genotype and time point as fixed factors followed by *post hoc* Tukey’s HSD test was conducted. Significance level for all tests was set at *p* < 0.05.

### Immunocytochemistry

Adult brains were dissected in ice-cold PBS and fixed immediately for 30–40 min in 4% paraformaldehyde. Fixed brains were blocked in 10% horse serum for 1 h at room temperature and 6–9 h at 4°C, followed by incubation with cocktail containing primary antibodies for 48 h. The primary antibodies used were anti-GFP (chicken, 1:2000, for GFP labeling and *CaLexA* measurements, Invitrogen #A10262, RRID: AB_2534023), anti-PDF (mouse, 1:5000, DSHB, PDF C7, RRID: AB_760350), anti-MYC (mouse, 1:1000, Cell Signaling Technology, #9B11, RRID: AB_2148465), anti-TH (rabbit, 1:1000, Invitrogen #P21962, RRID: AB_2539844), anti-GFP (mouse, 1:500, for GRASP, Sigma-Aldrich #G6539, RRID: AB_259941), and anti-PDF (rabbit, 1:30,000, M. Nitabach and T. C. Holmes). Following seven to eight serial washes with 0.5% Triton X-100 in PBS (0.5% PBT), brains were incubated with appropriate secondary antibodies for 24 h. Secondary antibodies were used at a concentration of 1:3000, and they were anti-chicken-Alexa Fluor 488 (Invitrogen, #A11039, RRID: AB_142924), anti-mouse-Alexa Fluor 546 (Invitrogen, #A11003, RRID: AB_141370), anti-mouse-Alexa Fluor 488 (Invitrogen, #A11001, RRID: AB_2534069), and anti-rabbit-Alexa Fluor 546 (Invitrogen, #A11035, RRID: AB_143051). Brains were washed and mounted on glass slides in 7:3 glycerol:PBS medium. Confocal images were taken on Zeiss LSM 880 (with Airyscan) microscope either with 20×, 40× (oil immersion), or 63× (oil immersion) objectives.

## Results

### PDFR signaling promotes wakefulness specifically during the day

**Figure 1.**
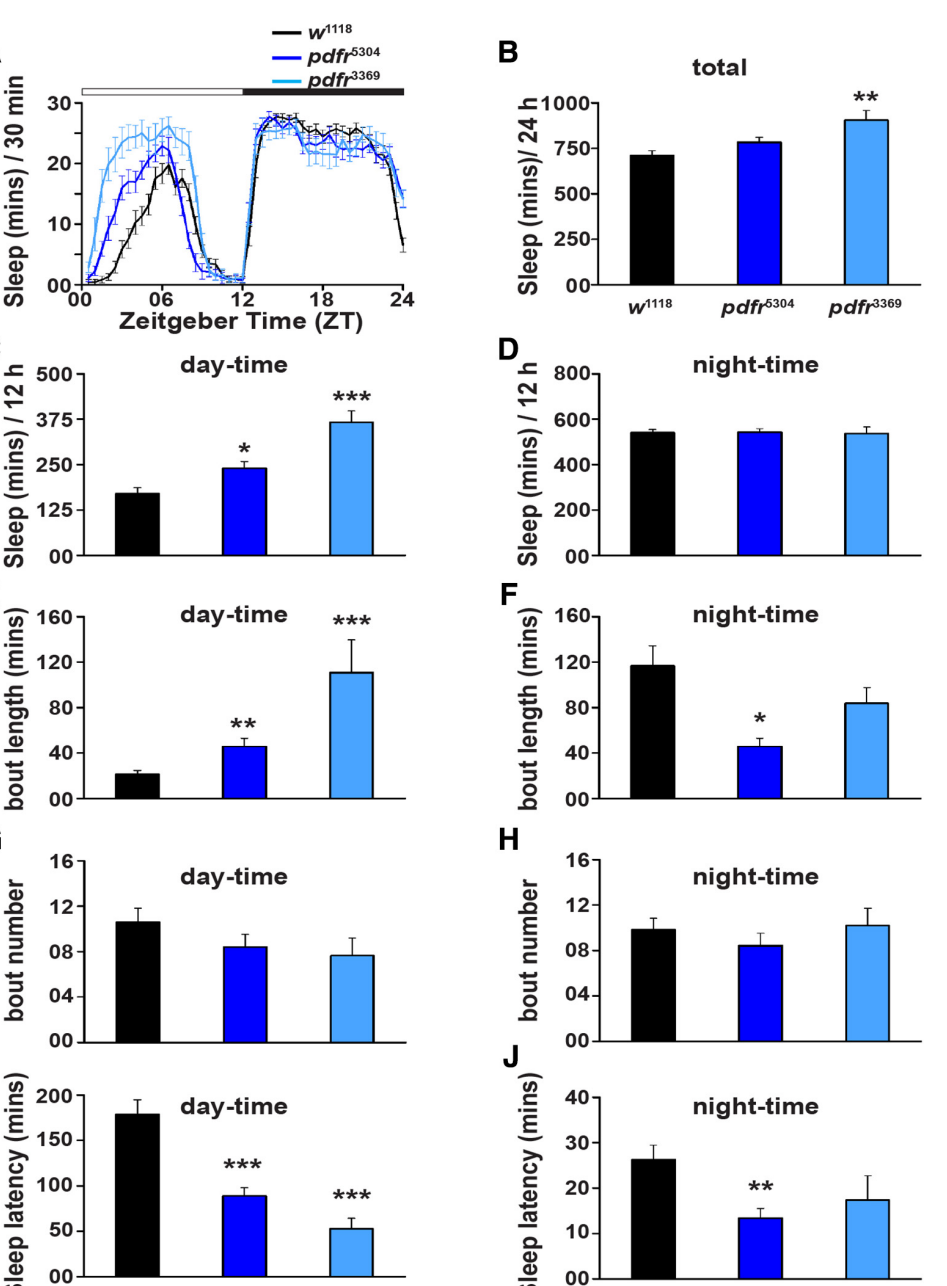
Loss-of-function mutants of *pdfr* display higher sleep duration during the day. ***A***, Amount of time spent sleeping estimated every 30 min as a function of time-of-day averaged across three cycles. Both *pdfr*^5304^ (*n* = 22 flies) and *pdfr*^3369^ (*n* = 14 flies) sleep more during the day time as compared to *w*^1118^ (*n* = 26 flies). Night-time sleep of *pdfr* mutants is similar to that of *w*^1118^ flies. White and black bars on top indicate 12 h of day and 12 h of night, respectively. ***B***, Total sleep over the 24-h cycle of *pdfr*^3369^ flies is significantly increased as compared to *w*^1118^ flies, whereas that of *pdfr*^5304^ is not different from *w*^1118^ flies. ***C***, Day-time sleep of both *pdfr* mutants is significantly higher than that of *w*^1118^ flies, whereas (***D***) night-time sleep of both *pdfr* mutants is similar to that of *w*^1118^ flies. ***E***, Average length of sleep bouts during the day is higher in both *pdfr* mutants as compared to *w*^1118^, while (***F***) average length of sleep bouts during the night in only *pdfr*^5304^ mutants is lower than that of *w*^1118^. Average number of sleep bouts of the *pdfr* mutants is comparable to that of *w*^1118^ both during (***G***) day and (***H***) night. Time taken to fall asleep (***I***) after lights-ON is lower in *pdfr*^5304^ and *pdfr*^3369^ mutants as compared to *w*^1118^ flies and (***J***) after lights-OFF is lower only in *pdfr*^5304^ as compared to *w*^1118^. Asterisks indicate levels of significance obtained from performing Students’ *t* tests for both mutants comparing each of them to *w*^1118^ independently; *p < 0.05, **p < 0.005, ***p < 0.0005. Error bars are SEM. Results representative from two independent experiments. See also Extended Data Figure 1-1.

To establish a phenotype on the basis of which, our screen to uncover downstream targets of PDFR could be designed, we examined two previously established loss-of-function mutants of the *pdfr* gene, *pdfr*^5304^ and *pdfr* ^3369^. A previous study had reported that both day-time and night-time sleep of these mutants is significantly higher than that of background control flies (Chung et al., 2009). However, we found that both mutants after backcrossing to the widely used *Iso31* (*w*^1118^) background for seven to eight generations exhibited significantly higher sleep only during the day-time under a standard LD12:12 cycle at 25°C (Fig. 1*A*,*C*; *w*^1118^ vs *pdfr*^5304^, Student’s two-tailed *t* test, *t*_(0.05,2,46)_ = -2.93, *p* < 0.05; *w*^1118^ vs *pdfr*^3369^, Student’s two-tailed *t* test, *t*_(0.05,2,38)_ = -6.33, *p* < 0.00001). Moreover, day-time sleep of *pdfr*^5304^ and *pdfr*^3369^ mutants was also different from one another (one-way ANOVA, *F*_(2,59)_ = 21.52, *p* < 0.00001), which was a peculiar observation seen in independent experiments. Functional analysis of the different domains that are deleted in these mutants, the former carries a deletion which excludes all the transmembrane domains and C terminus while the latter has a smaller deletion with loss of only one transmembrane domain and C terminus (Hyun et al., 2005) may explain why day-time sleep levels are different in the two mutants. Nonetheless, both mutants sleep much higher as compared to their background controls. Total sleep is significantly higher than the controls only in one of the mutants (Fig. 1*B*; *w*^1118^ vs *pdfr*^5304^, *t*_(0.05,2,46)_ = -1.92, *p* = 0.06; *w*^1118^ vs *pdfr*^3369^, *t*_(0.05,2,38)_ = -3.68, *p* < 0.005). However, night-time sleep of both the *pdfr* mutants was not different from that of the controls (Fig. 1*D*; *w*^1118^ vs *pdfr*^5304^, *t*_(0.05,2,46)_ = -0.09, *p* = 0.93; *w*^1118^ vs *pdfr*^3369^, *t*_(0.05,2,38)_ = 0.13, *p* = 0.9). These differences in sleep were not due to differences in activity levels (Extended Data Fig. 1-1*A*,*B*). Although the activity per waking minute is significantly lower for one of the mutants (Extended Data Fig. 1-1*B*; *w*^1118^ vs *pdfr*^5304^, *t*_(0.05,2,46)_ = 2.58, *p* < 0.05; *w*^1118^ vs *pdfr*^3369^, *t*_(0.05,2,38)_ = 1.91, *p* = 0.06) as compared to the control, this result was not seen in replicate experiments using the same genotypes (data not shown). Furthermore, the increase in day-time sleep seen in the *pdfr* mutants is also seen during subjective day time, when these flies are transferred to constant darkness (DD) at 25°C (Extended Data Fig. 1-1*C*,*D*; *w*^1118^ vs *pdfr*^5304^, *t*_(0.05,2,41)_ = -5.51, *p* < 0.00001; *w*^1118^ vs *pdfr*^3369^, *t*_(0.05,2,35)_ = -5.24, *p* < 0.00001). Moreover, we confirmed that back-crossing has not resulted in loss of the *pdfr* mutation by the observation that the behavioural phenotype of advanced evening activity peak (Hyun et al., 2005; Lear et al., 2005; Mertens et al., 2005) is reproduced under LD12:12 (Extended Data Fig. 1-1*A*). Taken together, these data suggest that absence of functional PDFR results in increased sleep duration specifically during the day.

10.1523/ENEURO.0129-18.2018.f1-1 Extended Data Figure 1-1 ***A***, Activity counts of male flies of *w*^1118^, *pdfr*^3369^, and *pdfr*^5304^ for every 15 min across time of the day shows that evening peak of *pdfr*^3369^ and *pdfr*^5304^ flies is advanced in phase as compared to that of *w*^1118^ flies. ***B***, Activity counts per waking minute of female flies whose sleep/wake patterns are described in Figure 1 is significantly lower in *pdfr*^5304^ flies as compared to *w*^1118^ flies in this particular run (not observed across independent runs). ***C***, Sleep per 30 min on first day of DD plotted against time of day and (***D***) amount of sleep in the first 12 h of first day in DD shows higher amount of sleep during subjective day time for *pdfr*^5304^ and *pdfr*^3369^ as compared to *w*^1118^ flies. Download Figure 1-1, TIF file.

While the quantity of day-time sleep has increased in the *pdfr* mutants, the quality of day-time sleep is also different as these *pdfr* mutants sleep longer within a typical sleep bout during the day time (Fig. 1*E*; *w*^1118^ vs *pdfr*^5304^, *t*_(0.05,2,46)_ = -3.23, *p* < 0.005; *w*^1118^ vs *pdfr*^3369^, *t*_(0.05,2,38)_ = -4.17, *p* < 0.0005). However, the number of such sleep bouts is not different in all three genotypes (Fig. 1*G*; *w*^1118^ vs *pdfr*^5304^, *t*_(0.05,2,46)_ = 1.27, *p* = 0.21; *w*^1118^ vs *pdfr*^3369^, *t*_(0.05,2,38)_ = 1.43, *p* = 0.16). These results suggest that sleep is more consolidated during the day time in the absence of functional *pdfr*. During the night, average sleep bout length is significantly lower in *pdfr*^5304^ than the control (Fig. 1*F*; *t*_(0.05,2,46)_ = 2.15, *p* < 0.05), whereas it is comparable to the control in the case of *pdfr*^3369^ (Fig. 1*F*; *t*_(0.05,2,38)_ = 1.26, *p* = 0.22), and number of sleep bouts during the night of both mutants is similar to that of the control (Fig. 1*H*; *w*^1118^ vs *pdfr*^5304^, *t*_(0.05,2,46)_ = -1.24, *p* = 0.22; *w*^1118^ vs *pdfr*^3369^, *t*_(0.05,2,38)_ = -0.2, *p* = 0.84). Interestingly, both mutants take lesser amount of time to fall asleep after lights-ON (Fig. 1*I*; *w*^1118^ vs *pdfr*^5304^, *t*_(0.05,2,46)_ = 4.16, *p* < 0.00001; *w*^1118^ vs *pdfr*^3369^, *t*_(0.05,2,38)_ = 5.3, *p* < 0.00001), whereas only *pdfr*^5304^ falls asleep sooner than *w*^1118^ after lights-OFF (Fig. 1*J*; *w*^1118^ vs *pdfr*^5304^, *t*_(0.05,2,46)_ = 3.18, *p* < 0.005; *w*^1118^ vs *pdfr*^3369^, *t*_(0.05,2,38)_ = 0.77, *p* = 0.45). Given that absence of *pdfr* leads to increased sleep duration as well as consolidated sleep and makes flies sleep sooner especially during the day time, these results corroborate the previously established role for PDFR signaling mediated by the PDF^+^ neurons in wake-promoting effects (Parisky et al., 2008; Shang et al., 2008; Sheeba et al., 2008; Chung et al., 2009) while highlighting a greater effect on day-time sleep compared to night.

### Screen for downstream targets of PDFR signaling

Previous studies that have characterized the expression pattern of *pdfr* using different antibodies against PDFR and/or promoter-mediated expression of cellular tags such as *myc* have revealed *pdfr* expression in a subset of circadian clock neurons, PI, EB, and ∼50 as yet non-characterized non-clock cells (Hyun et al., 2005; Lear et al., 2005; Mertens et al., 2005; Parisky et al., 2008; Im and Taghert, 2010). Therefore, on the basis of the predicted expression pattern of *pdfr* and potential sites in the vicinity of PDF projections, as well as those that function in sleep/wake regulation, we altered expression of *pdfr* in a total of 26 *GAL4* lines including distinct subsets of circadian clock neurons, MB, PI, central complex, and some neurotransmitter/peptide systems. Our interest was to identify driver lines whose targets responded with *both* an increase in day-time sleep on downregulation and a decrease in day-time sleep on overexpression (OEX) of *pdfr*. Moreover, to rule out non-specific effects on day-time sleep of either the *GAL4* or *UAS* parental line, we required the experimental flies to be significantly different as compared to both parental controls to be considered as a hit.

**Figure 2.**
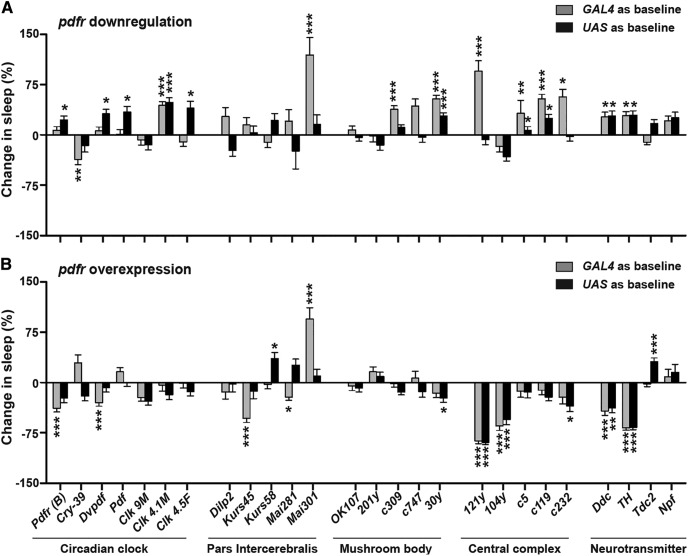
Screen to identify downstream targets of PDFR signaling. ***A***, Downregulation of *pdfr* using *UAS pdfr RNAi; UAS dcr* and (***B***) OEX of *pdfr* using *UAS pdfr* crossed with *GAL4* lines expressed in subsets of circadian clock neurons, PI, MB, central complex, and major neurotransmitter systems. Bars represent percentage increase (positive values) or decrease (negative values) in day-time sleep of experimental flies with respect to that of either *GAL4* (gray) and *UAS* (black) parental controls. Asterisks above the bars indicate level of significance when a one-way ANOVA with genotype as factor followed by *post hoc* Tukey’s test was done on raw day-time sleep levels. Several lines when used to downregulate *pdfr* show a significant increase in day-time sleep, but do not show a corresponding decrease in day-time sleep when *pdfr* is overexpressed (*Clk 4.1M GAL4*, *30y GAL4*, *c5 GAL4*, *c119 GAL4*), whereas a few lines show a significant decrease in day-time sleep when *pdfr* is overexpressed, but no corresponding increase in day-time sleep is seen when *pdfr* is downregulated (*121y GAL4*, *104y GAL4*). However, in two lines (*Ddc GAL4*, *TH GAL4*), when *pdfr* is downregulated, there occurs a significant increase in day-time sleep, and when *pdfr* is overexpressed, there occurs a significant decrease in day-time sleep. All other details are as in Figure 1. For all genotypes, *n* > 24 flies. Results representative from at least two independent experiments. See also Extended Data Figures 2-1, 2-2.

Quite surprisingly, downregulation and/or OEX in subsets of circadian clock neurons, which had previously been reported to modulate activity/rest rhythms in LD as well as in DD (Im and Taghert, 2010) did not show an effect on day-time sleep (Fig. 2). While downregulation of *pdfr* in ∼12-14 DN1_p_s using *Clk 4.1M GAL4* (Zhang et al., 2010) resulted in a significant increase in day-time sleep as compared to both parental controls (Fig. 2*A*; Extended Data Fig. 2-1), OEX of *pdfr* in the same subset of neurons did not result in a corresponding decrease in day-time sleep (Fig. 2*B*; Extended Data Fig. 2-2). Moreover, downregulation of *pdfr* in almost all PDFR^+^ clock neurons using *Pdfr (B) GAL4* (Im and Taghert, 2010) resulted in an increase in day-time sleep but this was significantly different only from the *UAS* parental control (Fig. 2*A*; Extended Data Fig. 2-1). OEX of *pdfr* using the same driver however resulted in significant decrease in day-time sleep only as compared to the *GAL4* control (Fig. 2*B*; Extended Data Fig. 2-2). Moreover, when *pdfr* was downregulated and/or overexpressed in a different combination of essentially the same cluster of circadian clock neurons (*Cry GAL4-39*; Klarsfeld et al., 2004), consistent effects on day-time sleep were not observed (Fig. 2). These results together lead to the interpretation that circadian clock neurons may not be major downstream targets of PDFR signaling that regulates day-time wakefulness.

10.1523/ENEURO.0129-18.2018.f2-1 Extended Data Figure 2-1 One-way ANOVA with genotype as fixed factor conducted for day-time sleep of flies with downregulation of *pdfr* in indicated drivers. F_(a-1), (N-k)_, where a is number of factor levels, *N* is the total number of replicates, and k refers to total number of groups. *F* statistic and *p* level of the main effect of genotype are indicated. Specific differences between genotypes determined after *post hoc* Tukey’s tests and indicated as asterisks in Figure 2*A*. Download Figure 2-1, DOCX file.

10.1523/ENEURO.0129-18.2018.f2-2 Extended Data Figure 2-2 One-way ANOVA with genotype as fixed factor conducted for day-time sleep of flies with OEX of *pdfr* in indicated drivers. F_(a-1), (N-k)_, where a is number of factor levels, N is the total number of replicates and k refers to total number of groups. *F* statistic and *p* level of the main effect of genotype are indicated. Specific differences between genotypes determined after *post hoc* Tukey’s tests and indicated as asterisks in Figure 2B. Download Figure 2-2, DOCX file.

In a recent study, it was found that PDF^+^ neurons communicate with DN1s, which then communicate with DH44^+^ PI neurons that brings about rhythmic locomotor activity under DD conditions (Cavanaugh et al., 2014). Given our finding that DN1s are most likely not the downstream targets of PDF^+^ neurons for sleep regulation, we next asked whether the PI neurons were direct recipients of PDF signals for modulation of day-time sleep. Barring a few non-specific parental effects on sleep, none of the 5 PI-specific *GAL4* drivers we screened showed any significant effects on day-time sleep when *pdfr* was downregulated and/or overexpressed (Fig. 2). Thus, although PI neurons appear to be well-placed to receive PDF signals, our finding suggests that they are not required for sleep regulation by the PDF^+^ neurons. In the light of our results and previous findings that PI neurons modulate sleep and wake levels (Foltenyi et al., 2007; Crocker et al., 2010), it appears that the PDF signaling and PI neurons are in different pathways of sleep and wake regulation.

Given that *pdfr* is expressed in the EB, and the suggestion that they could be the output neurons of PDF effects on sleep and wake levels (Parisky et al., 2008), we downregulated and overexpressed *pdfr* using *GAL4* drivers that distinctly label the EB. We found that downregulation of *pdfr* using *c119 GAL4* led to an increase in day-time sleep (Fig. 2A; Extended Data Fig. 2-1), however it was not accompanied with a corresponding decrease in day-time sleep on OEX of *pdfr* (Fig. 2*B*; Extended Data Fig. 2-2). Another *GAL4* driver targeting the EB did not show these effects on day-time sleep on downregulation and OEX of *pdfr* (c232 *GAL4*; Fig. 2). Thus, these results suggest that EB may not be downstream of PDFR signaling in sleep and wake modulation.

Im and Taghert (2010) reported that in addition to circadian clock neurons, PI, and EB, there are ∼50 cells in the brain that are PDFR^+^. We hypothesized that these 50 cells could potentially be any one of the MB and/or FB cells, neurons both of which are implicated in sleep regulation (Joiner et al., 2006; Pitman et al., 2006; Donlea et al., 2011; Donlea et al., 2014) and which may lie in the vicinity of projections of the PDF^+^ s-LN_v_ neurons. Not so surprisingly, none of the *GAL4* lines labeling either MB or FB showed significant and opposite effects on day-time sleep on downregulation and OEX of *pdfr* (Fig. 2). Interestingly, however, 4 *GAL4* drivers showed strong significant effects on day-time sleep on either downregulation only or OEX only of *pdfr*. Out of these, when *pdfr* was downregulated using the *30y GAL4* which labels the α/β lobes strongly and the rest of the MB weakly (Aso et al., 2009), day-time sleep was significantly higher as compared to both *GAL4* and *UAS* controls (Fig. 2*A*; Extended Data Fig. 2-1). Interestingly, OEX of *pdfr* using broader FB drivers such as *121y GAL4* and *104y GAL4* resulted in decrease of day-time sleep (Fig. 2*B*; Extended Data Fig. 2-2). However, similar results were not obtained with a more restricted driver (*c5 GAL4*) for FB thereby revealing non-specific effects of the OEX using the broad driver.

We next focused on a few neurotransmitter/peptide clusters that have previously been known to regulate sleep and wake such as dopamine, serotonin, octopamine and neuropeoptide F (NPF; Kume et al., 2005; Yuan et al., 2006; Crocker and Sehgal, 2008; He et al., 2013). To our surprise, when we downregulated *pdfr* in serotonergic and dopaminergic neurons using *Ddc GAL4*, as well as dopaminergic neurons alone using *TH GAL4*, we found that day-time sleep was significantly higher than the parental controls (Fig. 2*A*; Extended Data Fig. 2-1). Moreover, when we overexpressed *pdfr* using the same *GAL4* drivers, we found that day-time sleep was significantly lesser than the parental controls (Fig. 2*B*; Extended Data Fig. 2-2). However, there was no significant effect of either downregulating or overexpressing *pdfr* in either NPF^+^ or octopaminergic neurons on day-time sleep. Taken together, these results suggest that dopaminergic neurons are the most likely candidate for being the downstream targets of PDFR signaling to modulate day-time sleep and wake levels.

### PDFR signaling to dopaminergic neurons promotes day-time wakefulness

**Figure 3.**
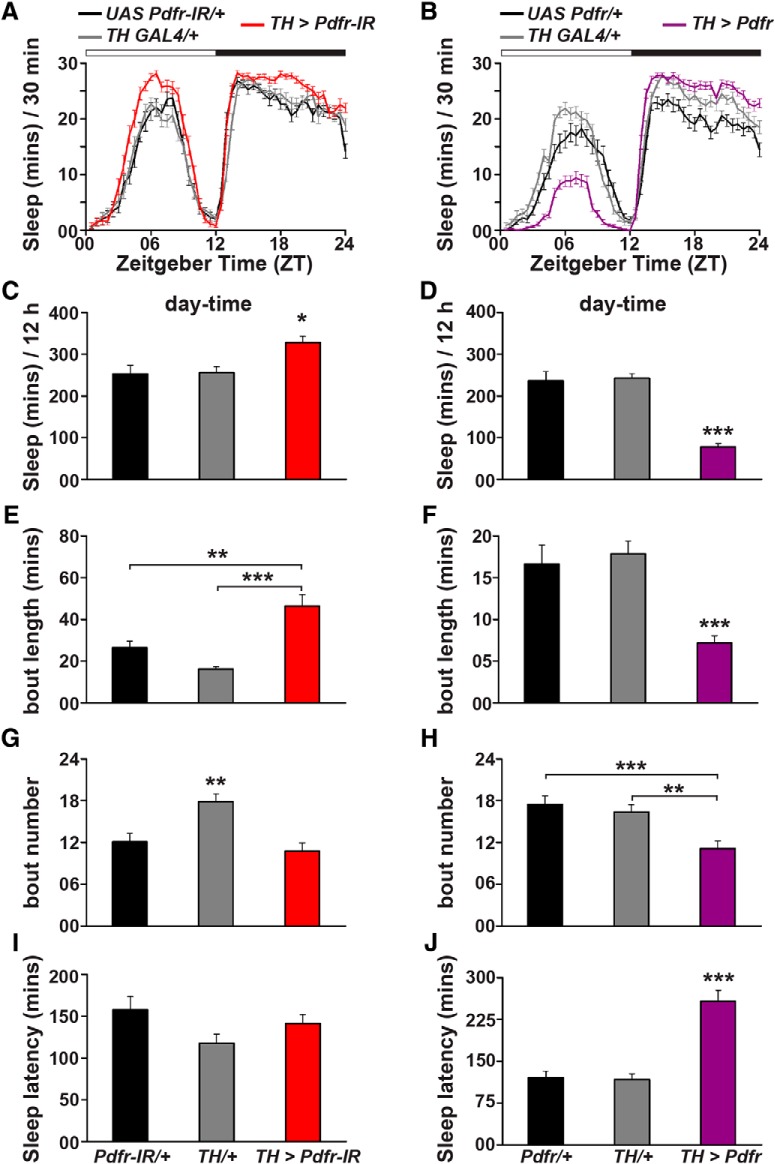
Quantity and quality of day-time sleep changes with changing *pdfr* expression levels in dopamine neurons. Sleep duration for every 30 min of an average LD12:12 cycle (***A***) when *pdfr* is downregulated and (***B***) when *pdfr* is overexpressed in dopaminergic neurons. ***A***, Day-time sleep as well as night-time sleep is increased in *TH GAL4 > UAS Pdfr RNAi; UAS dcr* (*n* = 32 flies) as compared to *TH GAL4/+* (*n* = 31 flies) and *UAS PDFR RNAi/+; UAS dcr/+* (*n* = 29 flies) controls, whereas (***B***) day-time sleep is decreased but night-time sleep is increased in *TH GAL4 > UAS Pdfr* (*n* = 28 flies) as compared to *TH GAL4/+* (*n* = 28 flies) and *UAS Pdfr/+* (*n* = 30 flies) controls. ***C***, Day-time sleep duration, (***E***) average sleep bout length, (***G***) average number of sleep bouts, and (***I***) latency to fall asleep after lights-ON for *TH GAL4 > UAS Pdfr RNAi; UAS dcr* flies compared to controls. ***D***, Day-time sleep duration, (***F***) average sleep bout length, (***H***) average sleep bout number, and (***J***) latency to fall asleep after lights-ON for *TH GAL4 > UAS Pdfr* flies compared to controls. Asterisks indicate significance levels obtained from one-way ANOVA with genotype as factor followed by *post hoc* Tukey’s test. All other details are as in Figure 1. Results representative from four independent experiments. See also Extended Data Figure 3-1.

We next examined the sleep/wake behavior of flies with downregulated or overexpressed *pdfr* in dopaminergic neurons in further detail. While downregulation (DR) of *pdfr* led to increase in day-time sleep and OEX of *pdfr* in dopaminergic neurons decreased day-time sleep (Fig. 3*A–D*; one-way ANOVA, DR, *F*_(2,89)_ = 6.53, *p* < 0.005; OEX, *F*_(2,88)_ = 43.81, *p* < 0.00001), interestingly both manipulations of *pdfr* expression levels led to an increase in night-time sleep (Fig. 3*A*,*B*; Extended Data Fig. 3-1*C*,*D*; DR, *F*_(2,89)_ = 12.48, *p* < 0.0005; OEX, *F*_(2,88)_ = 43.91, *p* < 0.00001). However, these differences in sleep levels were not as a result of changes in activity levels (Extended Data Fig. 3-1*A*,*B*; DR, *F*_(2,89)_ = 2.55, *p* = 0.08; OEX, *F*_(2,88)_ = 0.09, *p* = 0.9). Not only was the day-time sleep increased when *pdfr* was downregulated in dopaminergic neurons, the average sleep bout length was significantly longer as compared to both controls (Fig. 3*E*; *F*_(2,89)_ = 16.45, *p* < 0.00001), although the number of sleep bouts was not different from the *UAS* control (Fig. 3*G*; *F*_(2,89)_ = 10.33, *p* < 0.0005). Interestingly, the flies with downregulated *pdfr* in dopaminergic neurons took the same amount of time to fall asleep after lights-ON as the controls (Fig. 3*I*; *F*_(2,89)_ = 2.59, *p* = 0.08). Flies with overexpressed *pdfr* in dopaminergic neurons displayed shorter average sleep bouts during the day time (Fig. 3*F*; *F*_(2,88)_ = 13.78, *p* < 0.00001) as well as lesser number of such sleep bouts (Fig. 3*H*; *F*_(2,88)_ = 9.45, *p* < 0.0005). Unlike the *pdfr* downregulated flies, those with overexpressed *pdfr* in dopaminergic neurons took longer to fall asleep after lights-ON (Fig 3*J*; *F*_(2,88)_ = 28.96, *p* < 0.00001). Night-time sleep in both manipulations of *pdfr* expression levels was different from the controls only in terms of quantity, not in quality since sleep bout length and number were not affected (Extended Data Fig. 3-1*E–J*; sleep bout length: DR, *F*_(2,89)_ = 3.72, *p* < 0.05; OEX, *F*_(2,88)_ = 6.24, *p* < 0.005; sleep bout number: DR, *F*_(2,89)_ = 3.98, *p* < 0.05; OEX, *F*_(2,88)_ = 4.24, *p* < 0.05. Note that the significant values for E, G, and H are due to differences between experimental and only *UAS* control flies as shown in Extended Data Figure 3-1. Night sleep latency: DR, *F*_(2,89)_ = 1.1, *p* = 0.34; OEX, *F*_(2,88)_ = 2.81, *p* = 0.07). Thus, these results lead us to hypothesize that decreasing PDFR signaling to dopaminergic neurons increases day-time sleep, while increasing PDFR signaling to dopaminergic neurons suppresses day-time sleep and makes it fragmented, in addition to delaying sleep onset, suggesting that PDFR signaling to dopaminergic neurons is necessary for initiating and maintaining day-time wakefulness.

10.1523/ENEURO.0129-18.2018.f3-1 Extended Data Figure 3-1 Activity counts per waking minute of (***A***) *TH GAL4 > UAS Pdfr RNAi; UAS dcr* and (***B***) *TH GAL4 > UAS Pdfr* are not different from their respective parental controls. Night-time sleep of (***C***) *TH GAL4 > UAS Pdfr RNAi; UAS dcr* and (***D***) *TH GAL4 > UAS Pdfr* are significantly higher than their respective parental controls. Average length of sleep bout during night of (***E***) *TH GAL4 > UAS Pdfr RNAi; UAS dcr* is significantly higher than only *UAS Pdfr RNAi/+; UAS dcr/+* control flies, whereas (***F***) that of *TH GAL4 > UAS Pdfr* is significantly higher than both parental controls. Average number of sleep bouts during night of (***G***) *TH GAL4 > UAS Pdfr RNAi; UAS dcr* and (***H***) *TH GAL4 > UAS Pdfr* are significantly higher than only *UAS Pdfr RNAi/+; UAS dcr/+* and *UAS Pdfr* control flies, respectively. Sleep latency after lights-OFF of (***I***) *TH GAL4 > UAS Pdfr RNAi; UAS dcr* and (***J***) *TH GAL4 > UAS Pdfr* are not different from their respective parental controls. All other details are as in Figure 1. Download Figure 3-1, PDF file.

### PDFR^+^ PPM3 neurons modulate day-time wakefulness

**Figure 4.**
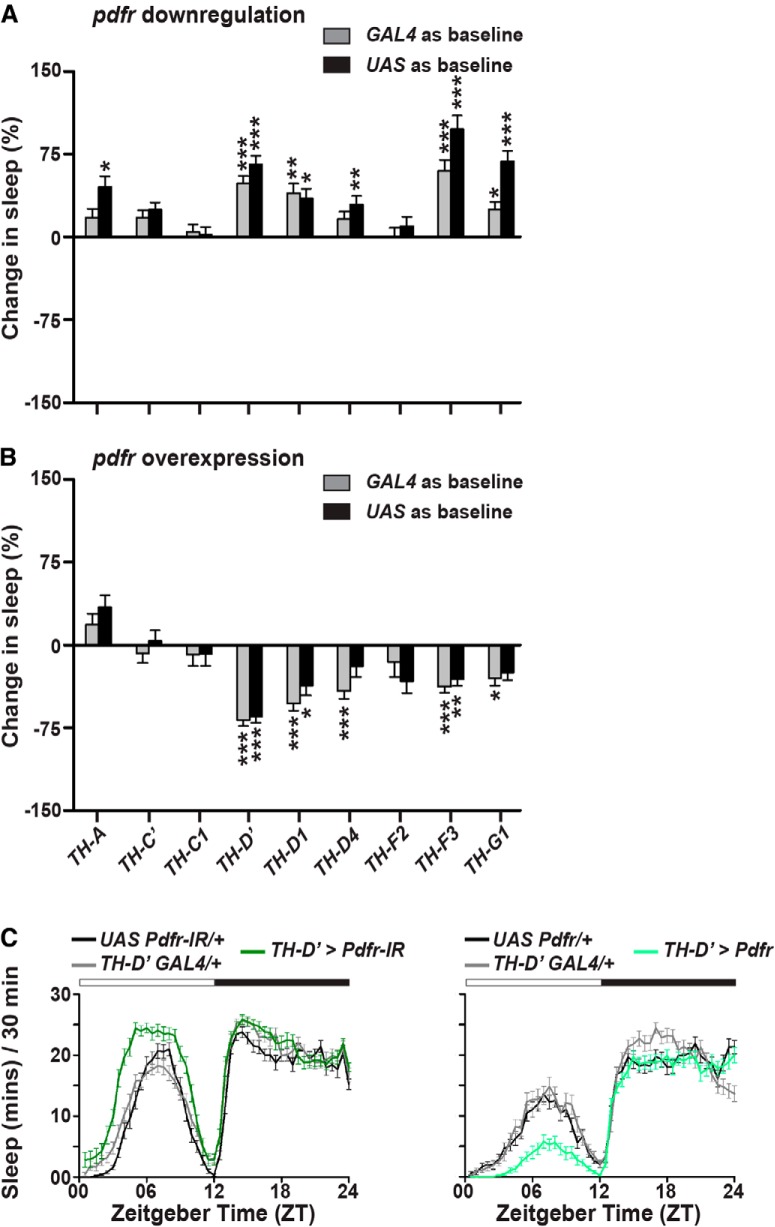
Screen to identify the subset of dopaminergic neurons that are downstream of PDFR signaling. ***A***, Downregulation of *pdfr* using *UAS pdfr RNAi; UAS dcr* and (***B***) OEX of *pdfr* using *UAS pdfr* crossed with *GAL4* lines expressed in different subsets of dopamine neurons. Downregulation and OEX of *pdfr* using only *TH-D’*, *TH-D1*, and *TH-F3 GAL4* lines leads to significant and opposite effects on day-time sleep. In all three lines, downregulation of *pdfr* leads to increase in day-time sleep, whereas OEX of *pdfr* leads to decrease in day-time sleep. For all genotypes, *n* > 24 flies. All other details are as in Figure 2. ***C***, Sleep duration for every 30 min of an average LD12:12 cycle of *TH-D’ GAL4> UAS Pdfr RNAi; UAS dcr* (*n* = 32 flies) compared to *TH-D’ GAL4/+* (*n* = 31 flies) and *UAS Pdfr RNAi/+; UAS dcr/+*(*n* = 31 flies) controls (left) and *TH-D’ GAL4> UAS Pdfr* (*n* = 32 flies) compared to *TH-D’ GAL4/+* (*n* = 32 flies) and *UAS Pdfr/+* (*n* = 32 flies) controls (right). Day-time sleep is increased with downregulation, whereas it is decreased with OEX of *pdfr* in a subset of dopaminergic neurons driven by *TH-D’ GAL4*. All other details are as in Figure 1. Results representative from at least two independent experiments. See also Extended Data Figures 4-1, 4-2, 4-3, 4-4, 4-5.

Dopaminergic neurons labeled on the basis of reactivity to antibody against Tyrosine Hydroxylase (anti-TH), which is the rate-limiting enzyme for dopamine synthesis, are divided into several subsets based on their anatomic location (Mao and Davis, 2009). There are two subsets present in the anterior brain (protocerebral anterior medial and lateral; PAM and PAL, respectively) and five subsets in the posterior brain (protocerebral posterior medial and lateral; PPM and PPL, respectively; PPM1-3 and PPL1-2). Of these, two previous studies have implicated a pair of bilaterally located PPL1 neurons (Liu et al., 2012) and a unilateral PPM3 neuron (Ueno et al., 2012) in promoting wakefulness through the inhibition of sleep-promoting FB. We asked whether the PDFR signaling is acting on either or both of these subsets to promote wakefulness specifically during the day. We used the previously created and characterized GAL4 drivers (*TH-A*, *C’*, *C1*, *D’*, *D1*, *D4*, *F2*, *F3*, and *G1*) targeting different subsets of dopaminergic neurons (Liu et al., 2012). When *pdfr* was downregulated or overexpressed using the *TH-A GAL4* which does not drive expression in any of the dopaminergic neurons (Liu et al., 2012), expectedly no difference in the day-time sleep levels was seen (Fig. 4*A*,*B*; Extended Data Figs. 4-1, 4-2), thus implying no non-specific *GAL4* effects. When we specifically targeted the downregulation or OEX to the anterior dopamine subsets PAM and PAL by using the *TH-C’* and *TH-C1 GAL4* drivers, no changes in day-time sleep were observed (Fig. 4*A*,*B*; Extended Data Figs. 4-1, 4-2), thus ruling out the involvement of PAM and PAL subsets in receiving PDFR signaling and promoting day-time sleep. The *TH-D*, *F* and *G* drivers are expressed in different subsets of PPM2, PPM3, PPL1 and PPL2 neurons (Extended Data Fig. 4-3; Liu et al., 2012). On applying the same stringent criteria as before, we find that downregulation and OEX of *pdfr* under the control of *TH-D’*, *D1*, and *F3* drivers result in significant and opposite changes in day-time sleep as compared to both parental controls (Fig. 4*A*,*C*; Extended Data Figs. 4-4, 4-1, 4-2). Thus, neurons belonging to PPL1, PPL2, and PPM3 subsets that are common to *TH-D’*, *D1*, and *F3* drivers but not expressed by *TH-D4*, *F2*, and *G1* drivers are the likely downstream targets of PDFR signaling important in modulating day-time wakefulness.

10.1523/ENEURO.0129-18.2018.f4-1 Extended Data Figure 4-1 One-way ANOVA with genotype as fixed factor conducted for day-time sleep of flies with downregulation of *pdfr* in indicated drivers. F_(a-1), (N-k)_, where a is number of factor levels, *N* is the total number of replicates, and k refers to total number of groups. *F* statistic and *p* level of the main effect of genotype are indicated. Specific differences between genotypes determined after *post hoc* Tukey’s tests and indicated as asterisks in Figure 4*A*. Download Figure 4-1, DOCX file.

10.1523/ENEURO.0129-18.2018.f4-2 Extended Data Figure 4-2 One-way ANOVA with genotype as fixed factor conducted for day-time sleep of flies with OEX of *pdfr* in indicated drivers. F_(a-1), (N-k)_, where a is number of factor levels, *N* is the total number of replicates, and k refers to total number of groups. *F* statistic and *p* level of the main effect of genotype are indicated. Specific differences between genotypes determined after *post hoc* Tukey’s tests and indicated as asterisks in Figure 4*B*. Download Figure 4-2, DOCX file.

10.1523/ENEURO.0129-18.2018.f4-3 Extended Data Figure 4-3 Expression of GFP under *TH GAL4*, *TH-D’*, *TH-D1*, *TH-D4*, *TH-F2*, *TH-F3*, *TH-G1 GAL4* drivers label different subsets of posterior protocerebrum lateral (PPL1-2) and medial (PPM1-3) neurons and their projections when posterior parts of the brains are imaged. Brains are costained with PDF for visualization of LN_v_ and their projections. Scale bars: 20 µm. Download Figure 4-3, PDF file.

10.1523/ENEURO.0129-18.2018.f4-4 Extended Data Figure 4-4 Sleep duration for every 30 min averaged across 3 d of LD12:12 cycles shows increased day-time sleep with downregulation (left) and decreased day-time sleep with OEX (right) of *pdfr* using both *TH-D1 GAL4* (top) and *TH-F3 GAL4* (bottom) drivers. For all genotypes, *n* > 24 flies. All other details are as in Figure 1. Download Figure 4-4, PDF file.

**Figure 5.**
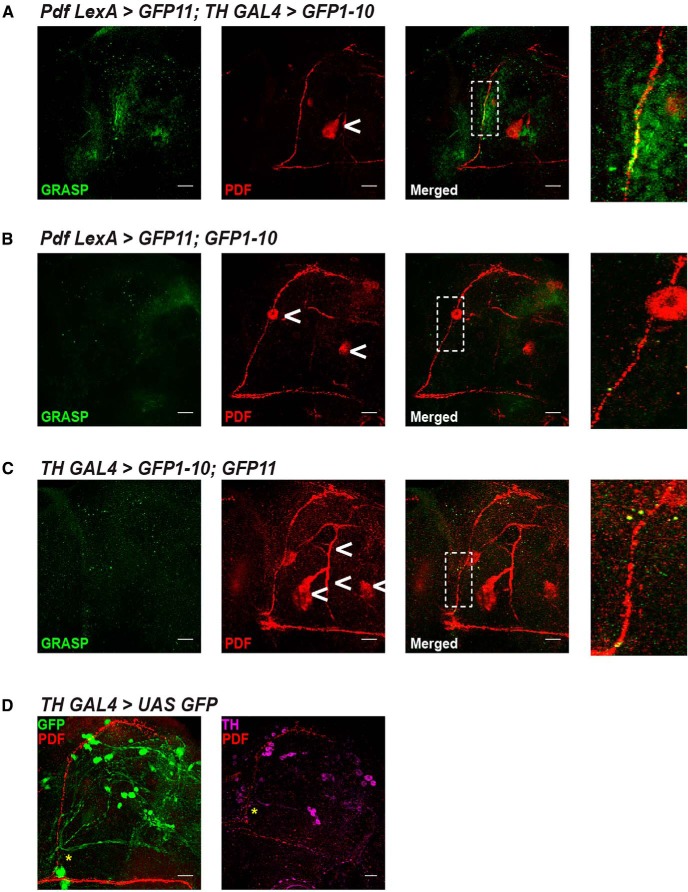
Anatomic connections between TH^+^ and PDF^+^ neurons. ***A***, Reconstituted GFP (GRASP) signal was detected in brains of flies expressing *LexAop CD4::spGFP11* under *Pdf LexA* control and *UAS CD4::spGFP1-10* under *TH GAL4* control (*n* = 22 hemispheres). GRASP signal colocalized with ascending portion of s-LN_v_ dorsal projections labeled with antibody against PDF. Highlighted region is magnified in the right-most panel. Control flies lacking expression of (***B***) *spGFP1-10* (*n* = 16 hemispheres) and (***C***) *spGFP11* (*n* = 16 hemispheres) do not show GRASP signal. Results representative from two independent experiments. Arrowheads indicate non-specific staining. Expression of GFP using *TH GAL4* and colabeling PDF (***D***, left) and using antibodies against TH and PDF (***D***, right) in wild-type flies reveal dopaminergic projection in the vicinity of ascending portion of s-LN_v_ dorsal projection as indicated by asterisks. Scale bar: 20 µm. See also Extended Data Figure 5-1.

10.1523/ENEURO.0129-18.2018.f4-5 Extended Data Figure 4-5 ***A***, *Pdfr myc* flies colabelled with antibodies against MYC and TH reveal TH^+^ and MYC^+^ cells to each other in the regions marked by the asterisks. ***B***, These regions contain TH^+^ neurons of the PPM3 subset (left), of which one neuron shows faint MYC^+^ signal and two neurons of the PPL1 subset (right) lie close to but do not overlap with MYC^+^ cell bodies. Scale bars: 20 µm. Download Figure 4-5, PDF file.

To identify the dopaminergic neurons that receive signals from PDF, we used the previously described *Pdfr myc* line (Im and Taghert, 2010) where *myc* is fused to the *Pdfr* gene, such that labeling MYC labels most PDFR^+^ neurons including clock neurons and ∼50 as yet uncharacterized non-clock neurons. We co-stained adult brains of *pdfr myc* flies with antibodies against TH and MYC and examined any overlap that may exist between TH^+^ and PDFR^+^ neurons. We found that one to two PDFR^+^ neurons always lie in the vicinity of PPL1 and PPM3 subset of dopaminergic neurons (Extended Data Fig. 4-5*A*; *n* = 22 hemispheres). On careful examination, we found that in three out of 11 brains visualized, there was one PPM3 neuron in each hemisphere that was both TH^+^ and PDFR^+^ (Extended Data Fig. 4-5*B*, left). The low number of brains showing TH^+^ and PDFR^+^ PPM3 neurons could be because of high background and low affinity of the anti-MYC antibody. However, in none of the brains could we detect any overlap between PPL1 TH^+^ and PDFR^+^ neurons, although they were quite close to each other (Extended Data Fig. 4-5*B*, right). Thus, we can only conclude that perhaps one PPM3 neuron per hemisphere may express the PDFR.

### PDF^+^ and TH^+^ neurons form synaptic contacts in sLN_v_ axons

Based on our results with altering *pdfr* levels that show that dopaminergic neurons are downstream targets of PDFR signaling, we next examined the nature of communication between PDF^+^ and dopaminergic neurons. Because PDF is a neuropeptide, it can have long-range non-synaptic effects on downstream neurons expressing the PDFR (Nässel and Winther, 2010). We conducted a GFP reconstitution across synaptic partners (GRASP) experiment which relies on two independent binary systems allowing the expression of two membrane-bound GFP fragments in different sets of neurons, such that GFP is reconstituted and fluoresces only when the fragments are present at synaptic distances (Gordon and Scott, 2009). A similar experiment done previously had shown the presence of synapses between PDF^+^ and dopaminergic neurons in the LN_v_ dendrites (Shang et al., 2011). However, here, we asked whether synapses between PDF^+^ and dopaminergic neurons occur specifically in the region of LN_v_ axons since we wished to examine postsynaptic targets of PDF. We costained adult brains of flies in which dopaminergic neurons expressed GFP1-10 fragment and PDF^+^ neurons expressed GFP11 fragment with anti-GFP antibody that specifically labels reconstituted GFP and anti-PDF to visualize the LN_v_ projections. We found that reconstituted GFP signal was specifically detected in the ascending part of the dorsal projection of sLN_v_ (Fig. 5*A*) which is an axonal process. However, when either fragment was individually driven in the PDF^+^ neurons or dopaminergic neurons alone, no GFP signal was detected (Fig. 5*B*,*C*) showing that the antibody does not recognize individual fragments of GFP. This shows that PDF^+^ and dopaminergic neurons form synaptic connections especially in the axonal projections of s-LN_v_, thus bolstering our finding that dopaminergic neurons are downstream of PDFR signaling. Furthermore, in brains with dopaminergic neurons labeled with promoter driven GFP (*TH GAL4 > UAS GFP*), as well as with anti-TH, we find a dopaminergic projection intersecting the ascending dorsal projection of s-LN_v_ (Fig. 5*D*, asterisk).

10.1523/ENEURO.0129-18.2018.f5-1 Extended Data Figure 5-1 ***A***, Sleep duration per 30 min is plotted against time of the day in LD12:12 of low (10 lux), moderate (300 lux), and high (2000 lux) light intensities. In moderate LD12:12 (middle), s^±^ L^±^ (*rpr*) flies sleep significantly more as compared to the s^+^ L^+^ (*GAL4*) control flies at almost all time points both during the day and night. s^-^ L^+^ (*Q128* and *NCQ128*) sleep similarly to their respective s^+^ L^+^ (*Q0* and *NCQ128*) controls during both day and night. s^H^ L^H^ and s^-^ L^H^ flies sleep significantly lesser than their controls, and s^H^ L^H^ flies sleep even lesser than the s^-^ L^H^ flies especially during the early part of the night. In both low (top) and high (bottom) LD12:12 cycles, s^H^ L^H^ flies take longer than all other genotypes including s^-^ L^H^ flies after lights-OFF to fall asleep. ***B***, In low (top) LD12:12 cycles, day-time sleep of s^±^ L^±^ flies is significantly higher than that of s^+^ L^+^ (*GAL4*) flies, whereas night-time sleep of s^±^ L^±^ flies is significantly higher than that of s^+^ L^+^ (*GAL4*) flies in moderate (middle) and high (bottom) LD12:12 cycles. In both low and moderate LD12:12 cycles, night-time sleep of s^H^ L^H^ and s^-^ L^H^ flies is significantly lesser than their respective controls, but not different from each other. Night-time sleep of s^H^ L^H^ is different from its respective control s^+^ L^+^ (*NCQ0*) as well as s^-^ L^H^ flies in high light intensity LD12:12 (bottom). For all genotypes, *n* > 24 flies. All other details are as in Figure 1. Download Figure 5-1, PDF file.

### Auxiliary role of sLN_v_ in mediating wake-promoting effects of l-LN_v_

While previous studies suggest a negligible role for the s-LN_v_ in the sleep/wake circuit (Shang et al., 2008; Chung et al., 2009), s-LN_v_ have been proposed to have a secondary role in promoting wake-mediating effects of l-LN_v_ (Parisky et al., 2008; Potdar and Sheeba, 2012). To explore their role further, we made use of previously reported toxic version of Huntingtin protein expression (*Htt Q128*, referred to as *Q128*, non-toxic form referred to as *Q0*) to selectively render s-LN_v_ dysfunctional (Sheeba et al., 2010) while simultaneously changing the electrical properties of the remaining l-LN_v_ by expressing the bacterial sodium channel *NachBac* (*NB*; Nitabach et al., 2006). We compared sleep patterns of the following genotypes of flies: those in which s-LN_v_ were dysfunctional but l-LN_v_ were normally firing (s^-^ L^+^ *Q128* and *NCQ128*), some neurons from both LN_v_ subsets were ablated (s^±^ L^±^ *rpr*, apoptotic gene *reaper*; Potdar and Sheeba, 2012), both LN_v_ subsets hyperexcited (s^H^ L^H^ *NBQ0*) and s-LN_v_ were dysfunctional but l-LN_v_ were hyperexcited (s^-^ L^H^ *NBQ128*) with their respective controls in which both LN_v_ were normally firing and functional (s^+^ L^+^ *Q0* for s^-^ L^+^ *Q128,* s^+^ L^+^ *GAL4* for s^±^ L^±^ *rpr,* s^+^ L^+^ *NCQ0* for s^H^ L^H^ *NBQ0* ands^-^ L^+^ *NCQ128*, *NC* refers to *dORKNC1*, which is a non-conducting potassium channel; Nitabach et al., 2002). Additionally, s^-^ L^+^ *NCQ128* served as control for s^-^ L^H^ *NBQ128*. Because l-LN_v_ wake-promoting effects are primarily mediated by light (Shang et al., 2008), we examined sleep levels of these flies in LD12:12 cycles with different day-time light intensities. We observe altered levels of day-time sleep only when the LN_v_ are ablated, but never when they are hyperexcited either completely or partially (Extended Data Fig. 5-1*A*,*B*; two-way ANOVA, 10 lux: *F*_(7,478)_ = 4.5, *p* < 0.0005; 300 lux: *F*_(7,468)_ = 8.27, *p* < 0.00001; 2000 lux: *F*_(7,476)_ = 13.91, *p* < 0.00001). Moreover, the increased levels of day-time sleep in s^±^ L^±^ flies is seen only when the light intensity is low (∼10 lux), but with increasing light intensity, day-time sleep levels are comparable to s^+^ L^+^ flies, suggesting that remaining LN_v_ that have not been ablated can modulate day-time sleep effectively especially in the presence of saturating light intensities (Extended Data Fig. 5-1*A*,*B*, left panels). Furthermore, day or night-time sleep levels are not altered when s-LN_v_ are dysfunctional and l-LN_v_ are normally firing (s^-^ L^+^). Interestingly, the finding that flies with hyperexcited LN_v_ show unchanged day-time sleep levels even in low light intensity LD12:12 cycles suggest that light-responsive l-LN_v_ can be saturated in terms of their firing capacity with as low light intensity as 10 lux. However, night-time sleep levels are always significantly lower than controls when both LN_v_ are hyperexcited (s^H^ L^H^; Extended Data Fig. 5-1*A*,*B*, right panels). Interestingly, night-time sleep levels of flies with dysfunctional s-LN_v_ but hyperexcited l-LN_v_ (s^-^ L^H^) is significantly reduced as compared to controls, but always higher than the s^H^ L^H^ flies (Extended Data Fig. 5-1*A*,*B*, right panels). In fact, under LD12:12 with low light intensity days (10 lux), we find that night-time sleep levels of s^-^ L^H^ flies are comparable with their s^+^ L^+^ controls (Extended Data Fig. 5-1*A*,*B*, right-top). These results validate that l-LN_v_ modulate wakefulness and further show that functional s-LN_v_ are required to mediate these effects.

### PDFR signaling inhibits PPM3 neuronal activity specifically during the day

Previous studies have shown that binding of PDF to PDFR results in a strong increase of cyclic AMP (cAMP) levels (Mertens et al., 2005; Shafer et al., 2008) and moderate increase of intracellular calcium (Ca^2+^) levels when expressed in HEK293 cells (Mertens et al., 2005). To test whether cAMP is the second messenger involved in mediating wakefulness through PDFR signaling, we overexpressed using *TH-F3 GAL4* either the catalytic or regulatory subunit of cAMP-dependent protein kinase A (PKA; *PKACA* and *PKAR*) which increases or reduces PKA activity, respectively. We found no significant changes in day-time sleep as a result of increasing or decreasing PKA activity (Extended Data Fig. 6-1*A*,*B*,*D*; one-way ANOVA, *F*_(2,86)_ = 0.44, *p* = 0.64), although day-time sleep of *TH-F3 GAL4 > UAS PKAR* flies was significantly lower as compared to only the *UAS PKAR* control flies (Extended Data Fig. 6-1*C*; one-way ANOVA, *F*_(2,77)_ = 4.41, *p* < 0.05). This shows that cAMP may not be the secondary messenger responding to PDFR signaling in the TH-F3^+^ neurons, as changing PKA activity levels has negligible effects on day-time sleep.

**Figure 6.**
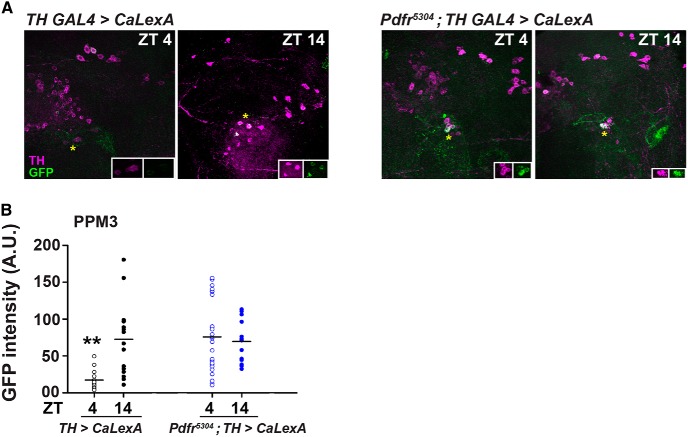
Intracellular Ca^2+^ levels in PPM3 neurons lower during day than night but remain similar during day and night in the absence of PDFR. ***A***, *TH GAL4* expressing *CaLexA* in *WT* and *pdfr* ^5304^ backgrounds costained with antibodies against TH to mark dopaminergic neurons and GFP to quantify intracellular Ca^2+^ levels at two time points: ZT4 and ZT14. *CaLexA*-driven GFP^+^ signal was detected at a low level at ZT4 whereas higher intensity at ZT14 (left panels) in *WT* background. A.U. = arbitrary units. *CaLexA*-driven GFP^+^ signal was detected at similar high level at ZT4 and ZT14 (right panels) in *pdfr* ^5304^ background. Asterisks indicate PPM3 neurons which are zoomed in inset. ***B***, Quantification of results in ***A*** shows significantly lower GFP fluorescence in *TH GAL4 > CaLexA* flies at ZT4 as compared to GFP fluorescence in *TH GAL4 > CaLexA* flies at ZT14, *pdfr* ^5304^; *TH GAL4 > CaLexA* flies at ZT4 and ZT14. All other details are as in Figure 1. See also Extended Data Figures 6-1, 6-2.

10.1523/ENEURO.0129-18.2018.f6-1 Extended Data Figure 6-1 ***A***, Sleep duration per 30 min is plotted against time of the day in LD12:12 for flies with either decreased (*TH-F3 GAL4 > UAS PKAR*) or increased (*TH-F3 GAL4 > UAS PKACA*) PKA signalling in TH-F3^+^ neurons. ***B***, Day-time sleep of *TH-F3 GAL4 > UAS PKAR* (*n* = 31 flies) is significantly lower than *UAS PKAR/+* (*n* = 22 flies) but not different from *TH-F3 GAL4/+* (*n* = 27 flies), whereas day-time sleep of *TH-F3 GAL4 > UAS PKACA* (*n* = 32 flies) is not different from both *UAS PKACA/+* (*n* = 30 flies) and *TH-F3 GAL4/+* (*n* = 27 flies). All other details are as in Figure 1. Download Figure 6-1, PDF file.

To assess the functional importance of the connectivity between PDF^+^ and dopaminergic neurons, we next examined intracellular Ca^2+^ levels in dopaminergic neurons at two time points, one during day [zeitgeber time (ZT)4; 4 h after lights-ON] and another during night (ZT14) in the presence and absence of functional PDFR (*Pdfr* ^5304^ mutant background). To quantify Ca^2+^ levels, we used the recently developed *CaLexA* method which relies on calcium-dependent-nuclear transport of *VP16:LexA* to drive GFP downstream of *LexAop* responder element (Masuyama et al., 2012). When the *CaLexA* transgenes are expressed using the broad dopaminergic driver *TH GAL4*, we find that one to two neurons of some dopaminergic subsets notably within the PAL, PPM2, and PPM3 clusters express GFP (Table 2). However, differential expression of GFP depending on time point and genotype is observed only in approximately one to two PPM3 neurons per hemisphere (Table 3). We find that in the presence of functional PDFR, at ZT4 when the levels of PDF are also high (Park et al., 2000), the amount of GFP^+^ signal seen in the PPM3 neurons is quite low (Fig. 6*A*,*B*; two-way ANOVA, *F*_(1,70)_ = 10.85, *p* < 0.005). However, at ZT14 when the levels of PDF are low (Park et al., 2000), amount of GFP^+^ signal seen in PPM3 neurons is significantly higher than that at ZT4 (Fig. 6*A*,*B*). In the absence of functional PDFR, at both ZT4 and ZT14, the GFP^+^ signal is high and not different from each other (Fig. 6*A*,*B*). Importantly, at ZT4, the GFP^+^ signal is significantly higher in the absence of functional PDFR than in its presence (Fig. 6). This shows that PDF acting on PDFR in the PPM3 dopaminergic neurons decreases Ca^2+^ levels specifically during the day time.

**Table 2.** Quantification of GFP positive cell numbers

Genotype	*TH GAL4 > UAS CaLexA*		*Pdfr^*5304*^; TH GAL4 > UAS CaLexA*	
Time point	ZT4	ZT14	ZT4	ZT14
*n* (hemispheres)	14	16	13	12
PAL (13)	1.4 ± 0.3	1.3 ± 0.3	2.4 ± 0.4	2.1 ± 0.4
PAM (5)	0.1 ± 0.09	0.4 ± 0.2	0.1 ± 0.1	0
PPM1 (5)^a^	0.1 ± 0.09	0	0	0
PPM2 (8)	2.1 ± 0.5	2.6 ± 0.4	2 ± 0.5	2 ± 0.4
PPM3 (8)	1.1 ± 0.3	1 ± 0.3	2. 2 ± 0.4	1.2 ± 0.3
PPL1 (12)	0.3 ± 0.1	0.7 ± 0.3	0.2 ± 0.1	1.5 ± 0.4
PPL2ab (6)	0.07 ± 0.07	0.8 ± 0.3	1.1 ± 0.3	0.8 ± 0.3
PPL2c (4)	0	0	0.08 ± 0.08	0.08 ± 0.08
PPL3 (1)	0.3 ± 0.2	0.3 ± 0.2	0.3 ± 0.3	0.2 ± 0.1
PPL4 (1)	0	0.06 ± 0.06	0	0

Number of GFP^+^ neurons as seen in different dopaminergic subsets (mean ± SEM) in brain hemispheres expressing *CaLexA* under the *TH GAL4* driver in wild-type and *pdfr* ^5304^ backgrounds during day (ZT4) and night (ZT14) time points. Numbers in parentheses indicate overall number of neurons of different subsets per hemisphere that are *TH GAL4*-positive as seen from data in Mao and Davis (2009). ^a^Number of neurons per brain.

**Table 3.** Quantification of GFP staining intensity as a proxy for calcium signalling and neuronal activity levels

Genotype	*TH GAL4 > UAS CaLexA*		*Pdfr^*5304*^; TH GAL4 > UAS CaLexA*	
Time point	ZT4	ZT14	ZT4	ZT14
*n* (hemispheres)	14	16	13	12
PAL	30.4 ± 4.8	32.3 ± 5.7	50.3 ± 5.9	36.8 ± 6
PAM	27.3 ± 13.8	55.2 ± 19.2	36.7 ± 10.6	0
PPM1	6.4 ± 0.3	0	0	0
PPM2	23.4 ± 5.2	41.3 ± 5.9	36.9 ± 6.7	23.8 ± 4.3
PPM3	17.2 ± 3	72.5 ± 12.1	75.8 ± 8.7	69.6 ± 7.6
PPL1	22 ± 9.9	17.4 ± 3.5	59.2 ± 41.3	22.6 ± 4.6
PPL2ab	15.4	20.2 ± 2.8	49.5 ± 10.9	36.7 ± 5.9
PPL2c	0	0	18.7	33.6
PPL3	6 ± 1.5	23.6 ± 8.6	11.4 ± 1.9	52.2 ± 0.6
PPL4	0	15.8	0	0

GFP^+^ fluorescence intensity (mean ± SEM) in different subsets of dopaminergic neurons.

Given that dopaminergic neurons are wake-promoting, inhibiting them should inhibit wakefulness. Yet, increasing PDFR in PPM3 neurons which, in accordance with our calcium level quantification should cause increased inhibition, results in decreased day-time sleep. Similarly, absence of *pdfr* leads to reduced inhibition (as seen from increased Ca^2+^ levels) of PPM3 neurons, yet behaviourally the flies sleep more during the day time. This indicates that PDF/PDFR signaling is acting on those PPM3 neurons that are, in effect, sleep-promoting. Thus, while a majority of dopaminergic neurons may be wake-promoting there may still be 1-2 PPM3 neurons which are PDFR^+^ and which effectively promote sleep. to examine this heterogeneity, we expressed the *Drosophila* temperature-sensitive cation channel *dTRPA1* (Hamada et al., 2008) in different subsets of dopaminergic neurons using *TH-D1*, *TH-D’* and *TH-F3 GAL4* drivers and examined the sleep levels of flies at a low ineffective temperature of 21°C as well as at a high activating temperature of 29°C. As reported in an earlier study (Liu et al., 2012), we find that flies sleep lesser both during the day and night when dopaminergic neurons driven by *TH-D1* and *TH-D’ GAL4* are hyperexcited (Extended Data Fig. 6-2; one-way ANOVA, *TH-D1*: *F*_(2,76)_ = 23.16, *p* < 0.00001; *TH-D’*: *F*_(2,78)_ = 28.88, *p* < 0.00001). However, when neurons expressed by the *TH-F3 GAL4* are hyperexcited, flies tend to sleep as much as their *GAL4* and *UAS* parental controls do, especially during the day time (Extended Data Fig. 6-2; one-way ANOVA, *F*_(2,85)_ = 2.29, *p* = 0.1). This can happen only if these neurons do not actually have any effect on sleep, or if they are a heterogeneous group of wake-promoting and sleep-promoting neurons, such that hyperexciting both leads to a cancellation of effects caused by both groups. Given that *TH-F3 GAL4*-driven dopaminergic neurons have effects on sleep when *pdfr* levels are altered, our results point toward the possibility of one to two PDFR^+^ PPM3 neurons that are also sleep-promoting.

10.1523/ENEURO.0129-18.2018.f6-2 Extended Data Figure 6-2 ***A***, Sleep duration per 30 min is plotted against time of the day in LD12:12 at a high temperature of 29°C for flies with hyperexcited dopamine neurons labelled by the *TH-D’*, *TH-D1*, and *TH-F3 GAL4* drivers. Both *TH-D’ GAL4 > UAS dTRPA1* (top) and *TH-D1 GAL4 > UAS dTRPA1* (middle) flies sleep lower both during the day and night as compared to their respective controls. *TH-F3 GAL4 > UAS dTRPA1* (bottom) do not differ in their sleep levels either during day or night as compared to both controls. For all genotypes, *n* > 30 flies. All other details are as in Figure 1. Download Figure 6-2, PDF file.

## Discussion

Dopamine is primarily involved in promoting wakefulness (Andretic et al., 2005; Kume et al., 2005) and is known to act on l-LN_v_ (Shang et al., 2011) as well as inhibit sleep-promoting dFB (Liu et al., 2012; Ueno et al., 2012) to carry out its wake-promoting function. Here, we uncover that certain dopamine neurons are in fact sleep-promoting and through the inhibitory action of PDFR signaling, wakefulness gets promoted specifically during the day. additional experiments that use optogenetic techniques can shed more light on whether these dopaminergic neurons promote sleep directly, or indirectly by preventing wakefulness either through a gating mechanism or by a permissive role. Interestingly, a previous study has found that dopamine acts on l-LN_v_ to promote wakefulness (Shang et al., 2011) and we find that PDFR signaling acts on dopamine neurons, suggesting a feed-forward pathway for wake promotion, where dopamine acting on l-LN_v_ promotes the inhibition of sleep-promoting dopaminergic neurons by PDFR signaling. The identity of dopamine neurons acting on l-LN_v_ and those responding to PDFR signaling may differ which can be uncovered with additional experiments.

The role of s-LN_v_ in modulating sleep and wake has been explored in some detail in the recent years. s-LN_v_ have also been shown to promote sleep via short NPF (sNPF) as well as myoinhibitory peptide (MiP) by inhibiting the wake-promoting l-LN_v_ (Shang et al., 2013; Oh et al., 2014). Here, we show that PDF^+^ s-LN_v_ make synaptic contacts with dopaminergic neurons (Fig. 5) and that PDFR signaling inhibits the downstream dopaminergic neurons (Fig. 6) to promote wakefulness during the day. Moreover, we have shown a secondary role for s-LN_v_ in modulating wake-promoting effects of l-LN_v_. Yet, how this wake-promoting signal which originates in the l-LN_v_ gets relayed to the s-LN_v_ is not understood. Furthermore, from our screen it is clear that this function is not mediated via PDFR signaling among the LN_v_, as downregulating and overexpressing *pdfr* in s-LN_v_ (*Clk 9M GAL4* and *Pdf GAL4*) do not result in any sleep defects. Thus, l-LN_v_ to s-LN_v_ wake-promoting signal is independent of PDF while s-LN_v_ to dopamine wake-promoting signal requires PDFR signaling.

PDFR being a class B1 GPCR utilizes cAMP as its second messenger (Shafer et al., 2008; Kunst et al., 2015), although there is evidence for Ca^2+^ also acting as the second messenger (Mertens et al., 2005). For most of the functions of PDF including stabilizing core clock proteins such as TIMELESS and PERIOD in different target neurons such as DN_1_s and s-LN_v_, cAMP is the major secondary messenger (Li et al., 2014; Seluzicki et al., 2014). Moreover, it is thought that different actions of PDF of slowing and speeding up of morning and evening clock neurons is also mediated by different components of cAMP signaling mechanism (Duvall and Taghert, 2012; Duvall and Taghert, 2013). However, here we show that for the function of regulating wake levels during the day time, PDFR signaling changes levels of intracellular Ca^2+^ in dopamine neurons with negligible role for cAMP signaling, suggesting a mechanism by which a neuropeptide that has diverse effects on its downstream targets can modulate different functions independently. We therefore identify a unique subset of downstream targets for PDFR signaling among the dopamine neurons that promote wakefulness depending on time of day.

Interestingly, in our screen we note that there are several driver lines using which there are significant changes in day-time sleep but with only one type of manipulation of *pdfr* levels (*Clk 4.1M*, *30y*, *104y*, *121y GAL4*). This may be due to ineffective downregulation of *pdfr* achieved through the *Pdfr RNAi* line with these particular drivers. Given that PDF is a neuropeptide which can have long-range non-synaptic effects (Nässel and Winther, 2010), even misexpressing it (*104y* and *121y GAL4*) in different substrates has resulted in altered day-time sleep levels. Because DH31 can also respond to PDFR (Kunst et al., 2015), it is possible that these effects could be mediated by DH31 binding to misexpressed PDFR. However, we find that this may not be the case as downregulating DH31-receptor in these regions does not cause changes in sleep levels (data not shown). Thus, we can conclude that in regions previously not known to express *pdfr*, misexpression of *pdfr* can cause sleep level deficits suggesting that PDF can act in regions which are not direct targets yet may lie in the vicinity of LN_v_ projections.

The role of PDF/PDFR signaling is well-known in synchronizing the free-running molecular rhythms in neurons across the circadian network (Peng et al., 2003; Lin et al., 2004; Wu et al., 2008; Yoshii et al., 2009). PDFR signaling in the “evening” neurons (LN_d_ and 5^th^ s-LN_v_) is important for appropriate phasing of the evening bout of activity in light/dark cycles (Yao and Shafer, 2014; Guo et al., 2016). While the role of PDF as a wake-signal has been known, here we demonstrate that a subset of dopaminergic neurons is downstream of the PDF/PDFR signaling. While the PDFR expression is not conclusive, we show that perhaps one PPM3 neuron per hemisphere may express the PDFR. Additional experiments that more directly test the functional connectivity between dopaminergic neurons and PDF^+^ neurons, as well as responsiveness of dopaminergic neurons to PDF may result in a clearer picture. Downregulating *pdfr* in these neurons results in increase of day-time sleep, which is a phenocopy of the sleep behavior of loss-of-function *pdfr* whole-body mutants. On the other hand, overexpressing *pdfr* in these neurons leads to decrease of day-time sleep specifically. We further show that PDF and dopaminergic neurons make synaptic contacts with each other at the site of the axonal projection of s-LN_v_. Moreover, the effect of PDFR signaling on the PPM3 neurons appears to be inhibitory, suggesting that the PDFR^+^ PPM3 neurons promote sleep. Taken together, we conclude that wake-promoting LN_v_ make synaptic connections with sleep-promoting dopaminergic neurons and promote wakefulness specifically during the day time through inhibitory PDFR signaling.

## Synthesis

Reviewing Editor: Rae Silver, Columbia University Decisions are customarily a result of the Reviewing Editor and the peer reviewers coming together and discussing their recommendations until a consensus is reached. When revisions are invited, a fact-based synthesis statement explaining their decision and outlining what is needed to prepare a revision will be listed below. The following reviewer(s) agreed to reveal their identity: Orie Shafer, Todd Holmes.

The reviewers of this paper, all experts, engaged in substantial discussion of its strengths and weaknesses, and came to complete agreement on its very substantial merits. In essence the work provides good evidence for the control of daytime wakefulness via the inhibition of identified sleep promoting dopaminergic neurons. This is a novel finding and one of great interest to the field. The rigor of the RNAi knockdown/overexpression screen is a strength of the experimental design and the study as a whole.

While the experiments are strong, there was some disagreement about the strength of the conclusions as well as some minor comments.

Suggestions regarding strength of the conclusions:

-The case for connectivity between PDF expressing clock neurons and the dopamine neurons could be stronger. A more direct assessment of physiological responsivity and connectivity might subsequently reveal a different picture. This point can be acknowledged in the discussion.

-Optogenetic experiments, while not required as a part of the current manuscript, would go a long ways to really nail the proposition that the PPM3 neurons are sleep promoting - as an alternative interpretation of the results, as these neurons may be playing a permissive or gating role.

-One criticism of the manuscript as currently written is that the authors interpret the failure to attain sleep phenotypes for RNAi mediated PdfR knockdown as support for the conclusion that the neurons in question do not regulate sleep in a PdfR dependent manner. It also seems plausible that the manipulation simply failed to knock-down the expression of the receptor. This can be acknowledged in the manuscript.

Minor:

-Does pdfr5304 differ from pdfr3369 for day sleep duration, and if so, why if they are backcrossed into the same genetic background? Please indicate how these mutants may differ if known.

-The extended data for several figures appear to be missing from the manuscript upload.

-The last figure (Figure 7) appears to be a Visual Abstract.

## Author Response

Dear Dr Silver,

We thank you and your reviewers for your positive review of our manuscript titled “Wakefulness is promoted during daytime by PDFR signalling to dopaminergic neurons in Drosophila melanogaster”. We are happy to note that you and your reviewers have found our manuscript potentially suitable for publication. We agree with the points raised by the reviewers and have now incorporated all the suggestions and comments in the revised version of the manuscript.

A detailed point-by-point response is given below and changes have been highlighted in the revised version.

1. Comments on the Visual Abstract for Author (Required):

The last figure of the manuscript (Figure 7) appears to be a good Visual Abstract. It is not clear is that is what the authors intended.

Our Response: We agree that it would be a useful visual abstract and now submit it as such.

2. Synthesis Statement for Author (Required):

The reviewers of this paper, all experts, engaged in substantial discussion of its strengths and weaknesses, and came to complete agreement on its very substantial merits. In essence the work provides good evidence for the control of daytime wakefulness via the inhibition of identified sleep promoting dopaminergic neurons. This is a novel finding and one of great interest to the field. The rigor of the RNAi knockdown/overexpression screen is a strength of the experimental design and the study as a whole.

Our Response: We thank the reviewers for the appreciation

3. While the experiments are strong, there was some disagreement about the strength of the conclusions as well as some minor comments.

Suggestions regarding strength of the conclusions:

-The case for connectivity between PDF expressing clock neurons and the dopamine neurons could be stronger. A more direct assessment of physiological responsivity and connectivity might subsequently reveal a different picture. This point can be acknowledged in the discussion.

Our Response: We agree that it would be helpful to have more direct assessment of physiological connections between PDF+ clock neurons and dopaminergic cells which we do not provide here. We acknowledge the same with the following sentence in the discussion on Page 18 Line 6.

“Further experiments that more directly test the functional connectivity between dopaminergic neurons and PDF+ neurons, as well as responsiveness of dopaminergic neurons to PDF may result in a clearer picture.”

4. -Optogenetic experiments, while not required as a part of the current manuscript, would go a long ways to really nail the proposition that the PPM3 neurons are sleep promoting - as an alternative interpretation of the results, as these neurons may be playing a permissive or gating role.

Our Response: We agree that optogenetic experiments are likely to provide evidence for the role of PPM3 neurons in sleep. However, in the absence of such evidence we discuss the alternate possibilities namely their permissive or gating role on Page 16 Line 5.

“Further experiments that utilize optogenetic techniques can shed more light on whether these dopaminergic neurons promote sleep directly, or indirectly by preventing wakefulness either through a gating mechanism or by a permissive role.”

5. -One criticism of the manuscript as currently written is that the authors interpret the failure to attain sleep phenotypes for RNAi mediated PdfR knockdown as support for the conclusion that the neurons in question do not regulate sleep in a PdfR dependent manner. It also seems plausible that the manipulation simply failed to knock-down the expression of the receptor. This can be acknowledged in the manuscript.

Our Response: We agree that an alternate explanation for the failure to detect a sleep phenotype with RNAi may be that the method was inefficient and we note the same in discussion on Page 17.

“This may be due to ineffective downregulation of pdfr achieved through the Pdfr RNAi line with these particular drivers.”

Minor:

-Does pdfr5304 differ from pdfr3369 for day sleep duration, and if so, why if they are backcrossed into the same genetic background? Please indicate how these mutants may differ if known.

Our Response: We regret the lack of clarity and provide the information on Page 5.

“Moreover, daytime sleep of pdfr5304 and pdfr3369 mutants was also different from one another (one-way ANOVA, F2, 59 = 21.52, p &lt; 0.00001), which was a peculiar observation seen in independent experiments. Functional analysis of the different domains that are deleted in these mutants - the former carries a deletion which excludes all the transmembrane domains and C-terminus while the latter has a smaller deletion with loss of only one transmembrane domain and C-terminus (Hyun et al., 2005) may explain why day-time sleep levels are different in the two mutants. Nonetheless, both mutants sleep much higher as compared to their background controls.”

-The extended data for several figures appear to be missing from the manuscript upload.

Our Response: We regret the inconvenience and will rectify the loss of information and will attempt to rectify the error with the help of journal staff.

-The last figure in the manuscript (Figure 7) appears to be a Visual Abstract.

Our Response: We will provide the same as a visual abstract. We thank the reviewers for the suggestion.
