# Supplementary figures and images for: Wakefulness Is Promoted during Day Time by PDFR Signalling to Dopaminergic Neurons in Drosophila melanogaster
Source: eNeuro. 2018 Aug 8;5(4):ENEURO.0129-18.2018. doi: 10.1523/ENEURO.0129-18.2018 (PMC6102377; doi:10.1523/ENEURO.0129-18.2018)

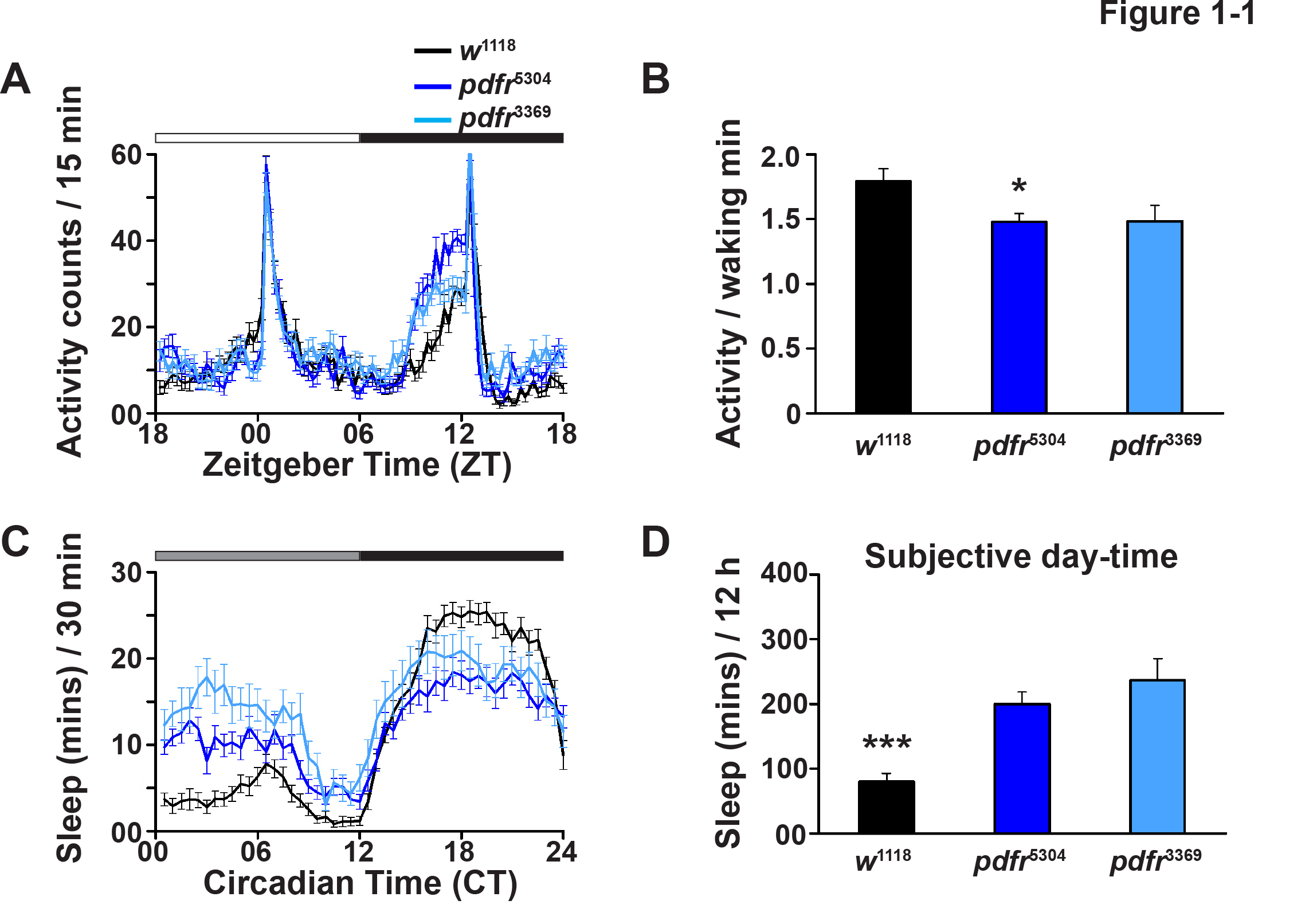

Supplement: Extended Data Figure 1-1 — A, Activity counts of male flies of w1118, pdfr3369, and pdfr5304 for every 15 min across time of the day shows that evening peak of pdfr3369 and pdfr5304 flies is advanced in phase as compared to that of w1118 flies. B, Activity counts per waking minute of female flies whose sleep/wake patterns are described in Figure 1 is significantly lower in pdfr5304 flies as compared to w1118 flies in this particular run (not observed across independent runs). C, Sleep per 30 min on first day of DD plotted against time of day and (D) amount of sleep in the first 12 h of first day in DD shows higher amount of sleep during subjective day time for pdfr5304 and pdfr3369 as compared to w1118 flies. Download Figure 1-1, TIF file. [file sup_enu-eN-NWR-0129-18-s03.tif]

Figure 3-1

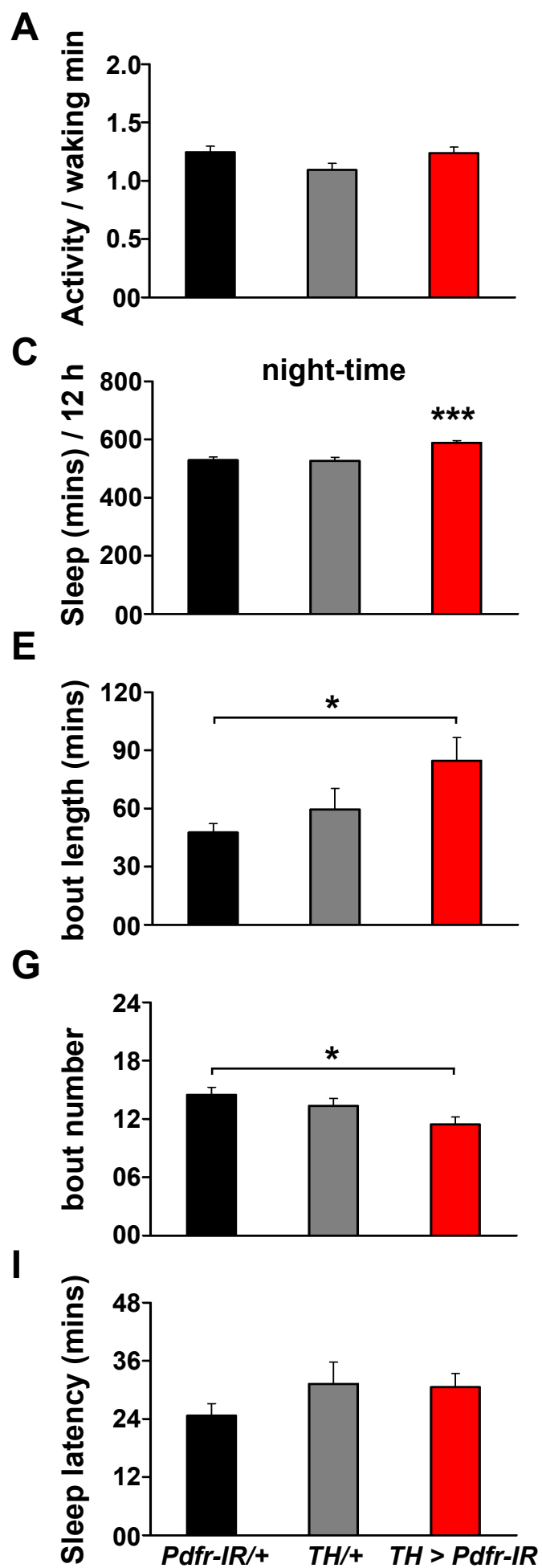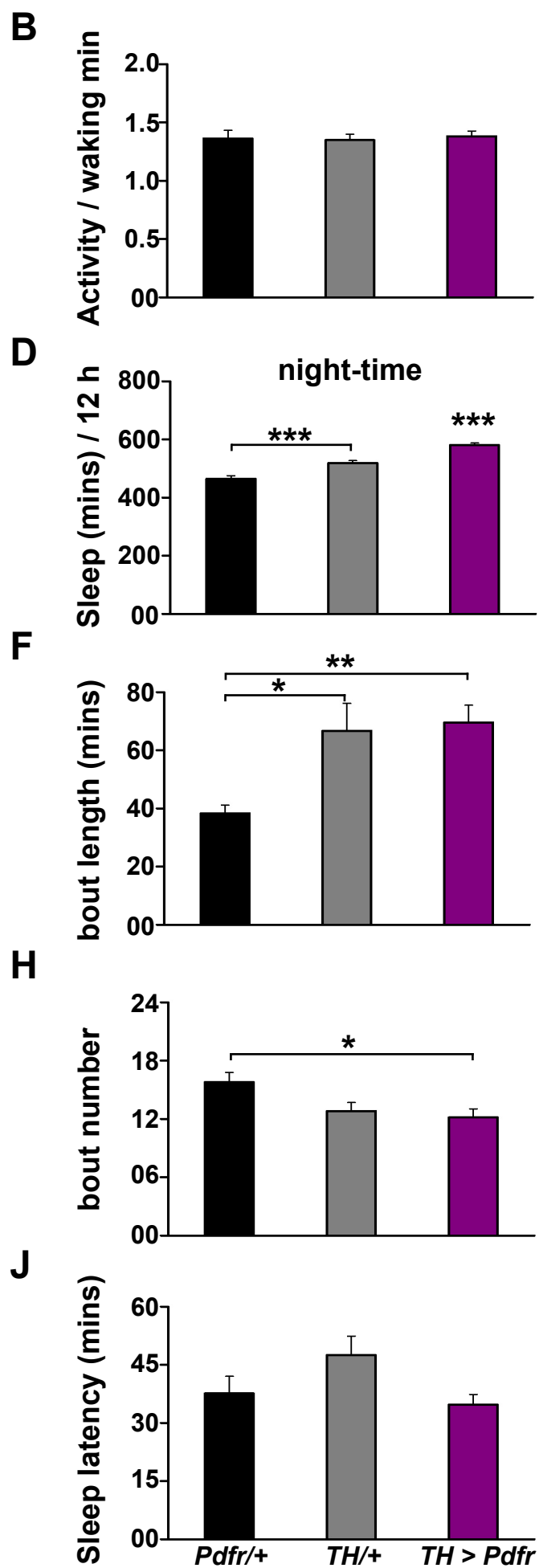

Supplement: Extended Data Figure 3-1 — Activity counts per waking minute of (A) TH GAL4 > UAS Pdfr RNAi; UAS dcr and (B) TH GAL4 > UAS Pdfr are not different from their respective parental controls. Night-time sleep of (C) TH GAL4 > UAS Pdfr RNAi; UAS dcr and (D) TH GAL4 > UAS Pdfr are significantly higher than their respective parental controls. Average length of sleep bout during night of (E) TH GAL4 > UAS Pdfr RNAi; UAS dcr is significantly higher than only UAS Pdfr RNAi/+; UAS dcr/+ control flies, whereas (F) that of TH GAL4 > UAS Pdfr is significantly higher than both parental controls. Average number of sleep bouts during night of (G) TH GAL4 > UAS Pdfr RNAi; UAS dcr and (H) TH GAL4 > UAS Pdfr are significantly higher than only UAS Pdfr RNAi/+; UAS dcr/+ and UAS Pdfr control flies, respectively. Sleep latency after lights-OFF of (I) TH GAL4 > UAS Pdfr RNAi; UAS dcr and (J) TH GAL4 > UAS Pdfr are not different from their respective parental controls. All other details are as in Figure 1. Download Figure 3-1, PDF file. [file sup_enu-eN-NWR-0129-18-s04.pdf]

Figure 4-4

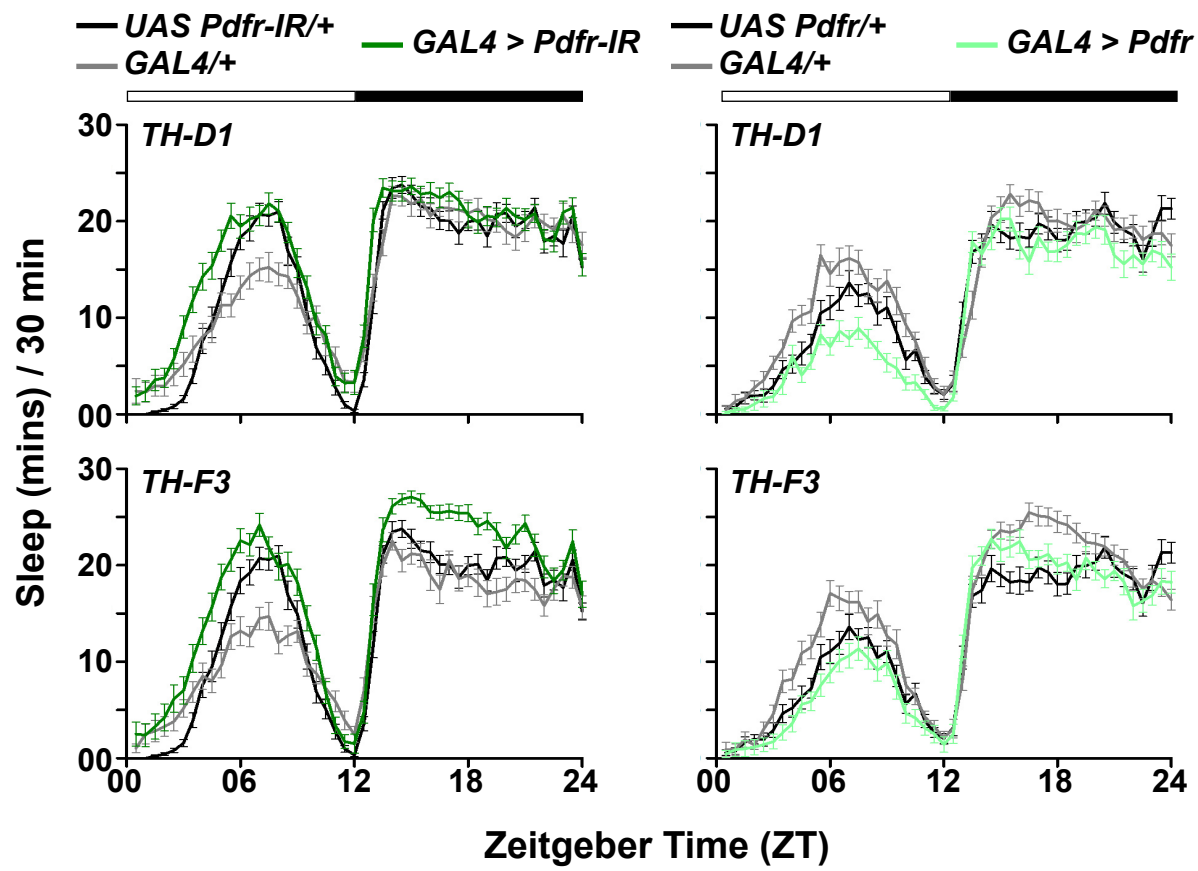

Supplement: Extended Data Figure 4-4 — Sleep duration for every 30 min averaged across 3 d of LD12:12 cycles shows increased day-time sleep with downregulation (left) and decreased day-time sleep with OEX (right) of pdfr using both TH-D1 GAL4 (top) and TH-F3 GAL4 (bottom) drivers. For all genotypes, n > 24 flies. All other details are as in Figure 1. Download Figure 4-4, PDF file. [file sup_enu-eN-NWR-0129-18-s06.pdf]

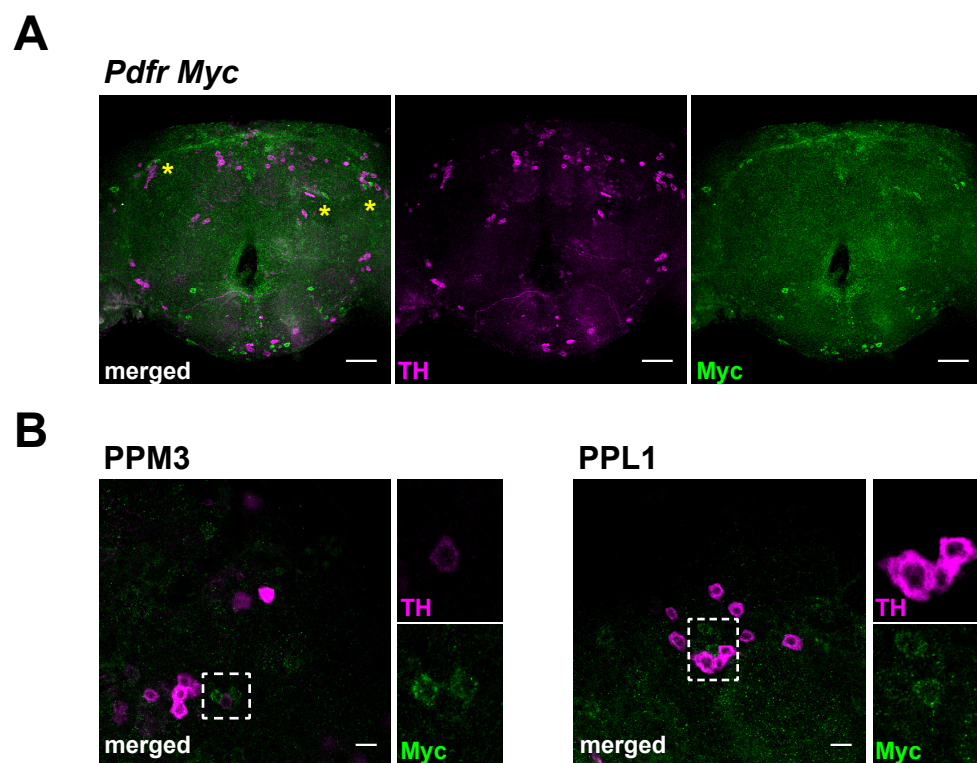

Supplement: Extended Data Figure 4-5 — A, Pdfr myc flies colabelled with antibodies against MYC and TH reveal TH+ and MYC+ cells to each other in the regions marked by the asterisks. B, These regions contain TH+ neurons of the PPM3 subset (left), of which one neuron shows faint MYC+ signal and two neurons of the PPL1 subset (right) lie close to but do not overlap with MYC+ cell bodies. Scale bars: 20 µm. Download Figure 4-5, PDF file. [file sup_enu-eN-NWR-0129-18-s07.pdf]

Figure 5-1

**A**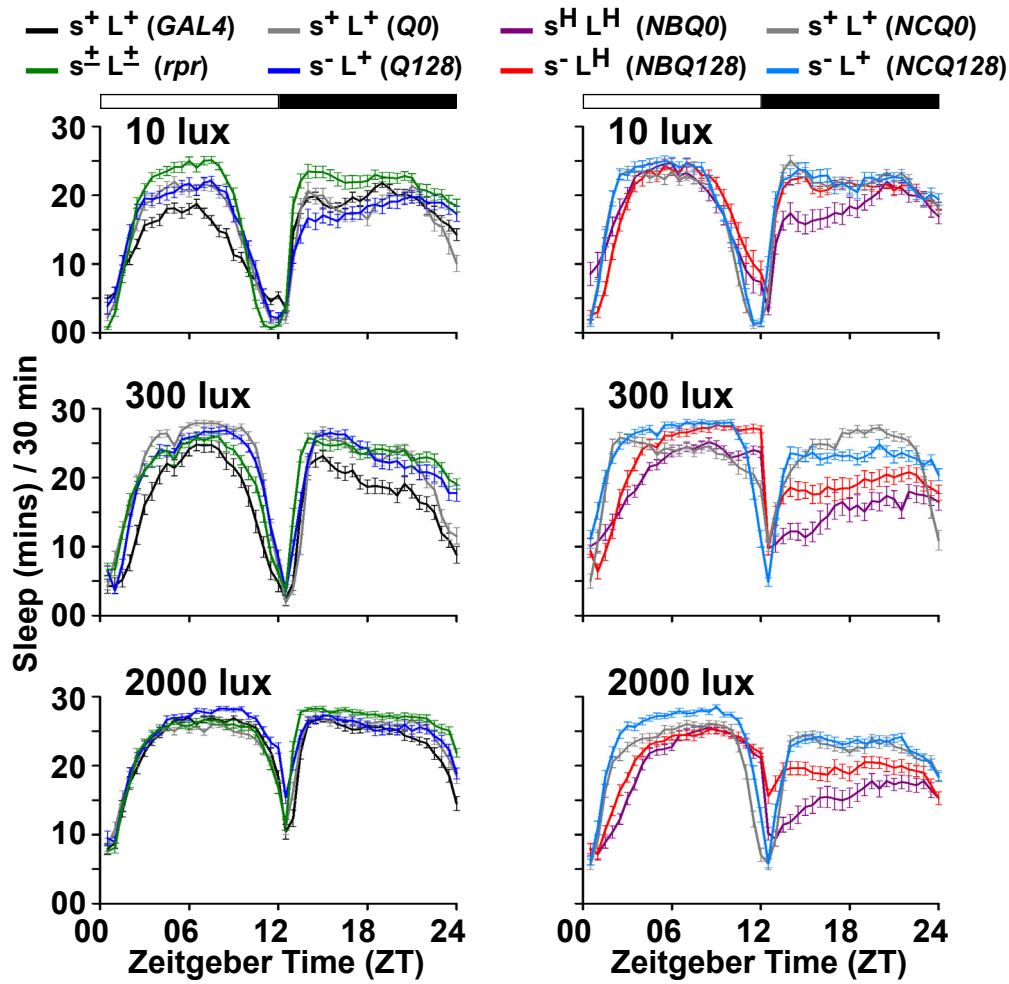**B**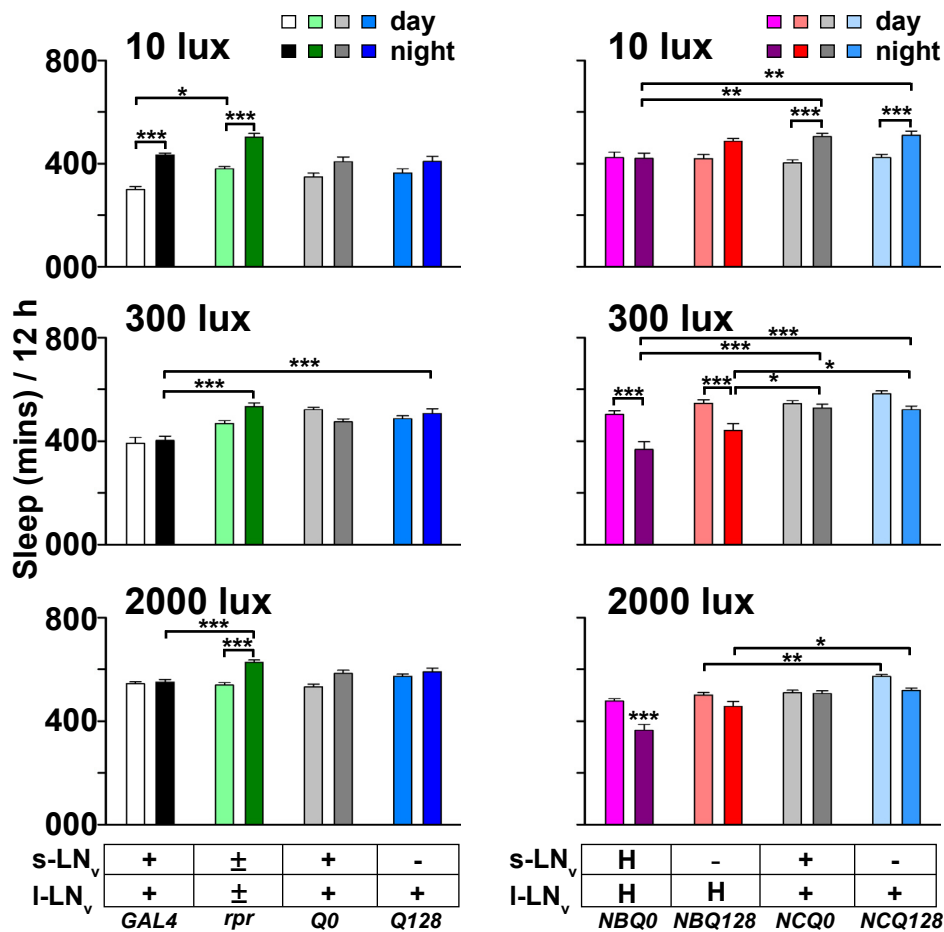

Supplement: Extended Data Figure 5-1 — A, Sleep duration per 30 min is plotted against time of the day in LD12:12 of low (10 lux), moderate (300 lux), and high (2000 lux) light intensities. In moderate LD12:12 (middle), s± L± (rpr) flies sleep significantly more as compared to the s+ L+ (GAL4) control flies at almost all time points both during the day and night. s- L+ (Q128 and NCQ128) sleep similarly to their respective s+ L+ (Q0 and NCQ128) controls during both day and night. sH LH and s- LH flies sleep significantly lesser than their controls, and sH LH flies sleep even lesser than the s- LH flies especially during the early part of the night. In both low (top) and high (bottom) LD12:12 cycles, sH LH flies take longer than all other genotypes including s- LH flies after lights-OFF to fall asleep. B, In low (top) LD12:12 cycles, day-time sleep of s± L± flies is significantly higher than that of s+ L+ (GAL4) flies, whereas night-time sleep of s± L± flies is significantly higher than that of s+ L+ (GAL4) flies in moderate (middle) and high (bottom) LD12:12 cycles. In both low and moderate LD12:12 cycles, night-time sleep of sH LH and s- LH flies is significantly lesser than their respective controls, but not different from each other. Night-time sleep of sH LH is different from its respective control s+ L+ (NCQ0) as well as s- LH flies in high light intensity LD12:12 (bottom). For all genotypes, n > 24 flies. All other details are as in Figure 1. Download Figure 5-1, PDF file. [file sup_enu-eN-NWR-0129-18-s08.pdf]

Figure 6-1

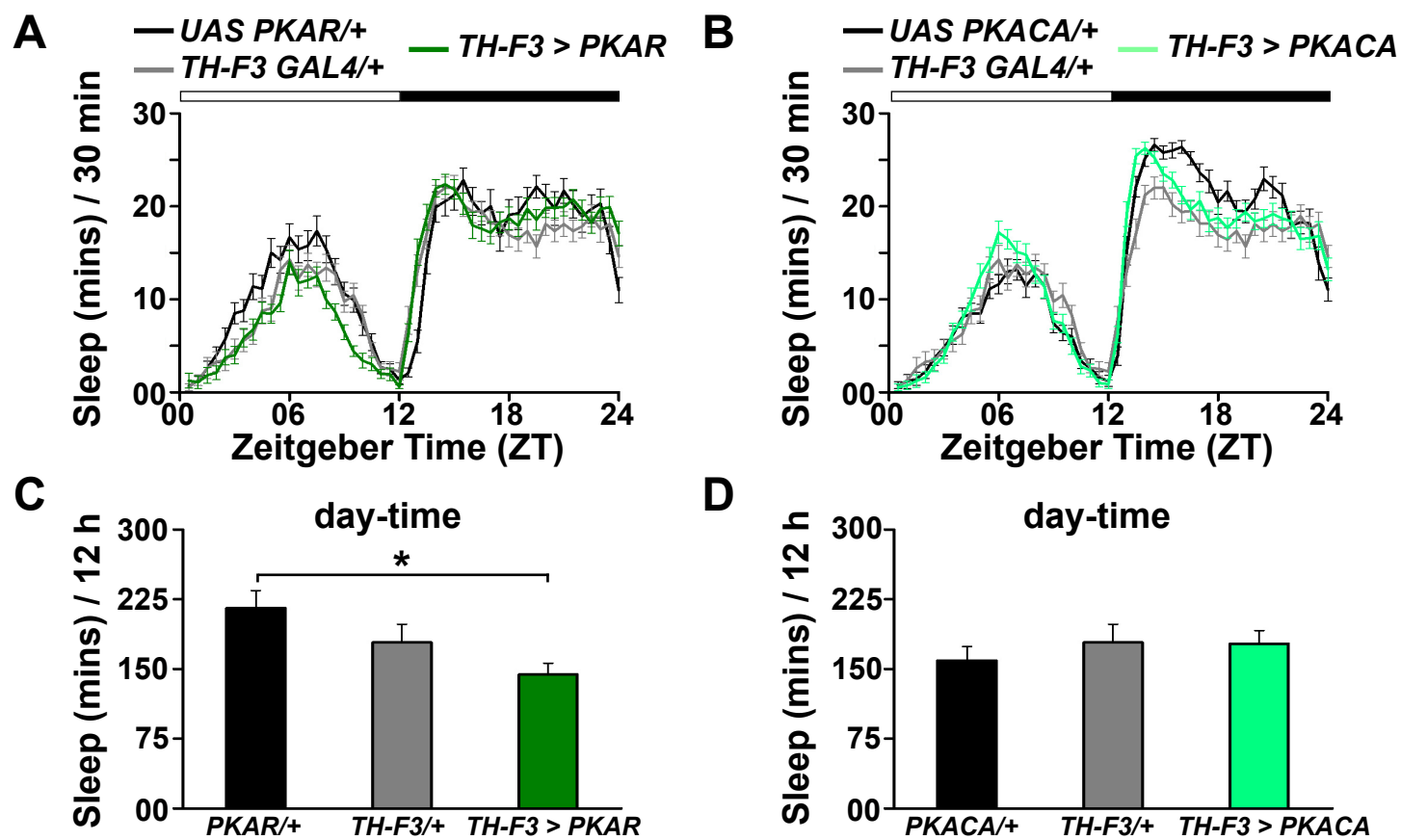

Supplement: Extended Data Figure 6-1 — A, Sleep duration per 30 min is plotted against time of the day in LD12:12 for flies with either decreased (TH-F3 GAL4 > UAS PKAR) or increased (TH-F3 GAL4 > UAS PKACA) PKA signalling in TH-F3+ neurons. B, Day-time sleep of TH-F3 GAL4 > UAS PKAR (n = 31 flies) is significantly lower than UAS PKAR/+ (n = 22 flies) but not different from TH-F3 GAL4/+ (n = 27 flies), whereas day-time sleep of TH-F3 GAL4 > UAS PKACA (n = 32 flies) is not different from both UAS PKACA/+ (n = 30 flies) and TH-F3 GAL4/+ (n = 27 flies). All other details are as in Figure 1. Download Figure 6-1, PDF file. [file sup_enu-eN-NWR-0129-18-s09.pdf]

Figure 6-2

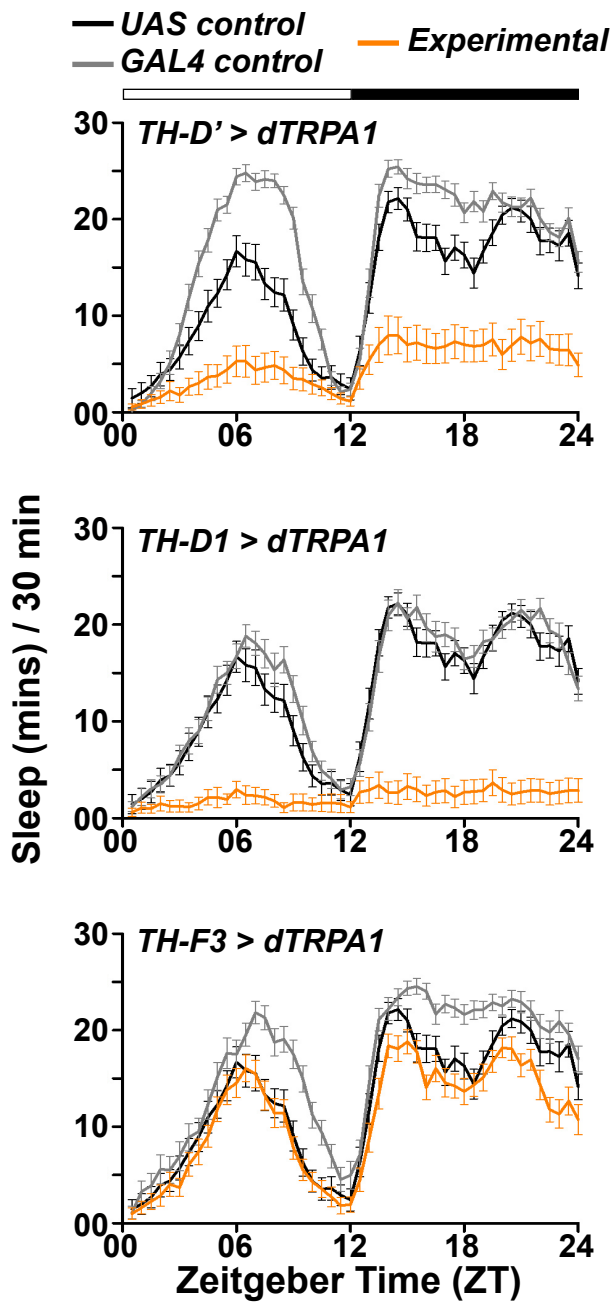

Supplement: Extended Data Figure 6-2 — A, Sleep duration per 30 min is plotted against time of the day in LD12:12 at a high temperature of 29°C for flies with hyperexcited dopamine neurons labelled by the TH-D’, TH-D1, and TH-F3 GAL4 drivers. Both TH-D’ GAL4 > UAS dTRPA1 (top) and TH-D1 GAL4 > UAS dTRPA1 (middle) flies sleep lower both during the day and night as compared to their respective controls. TH-F3 GAL4 > UAS dTRPA1 (bottom) do not differ in their sleep levels either during day or night as compared to both controls. For all genotypes, n > 30 flies. All other details are as in Figure 1. Download Figure 6-2, PDF file. [file sup_enu-eN-NWR-0129-18-s10.pdf]
