# Supplementary material for: Wakefulness Is Promoted during Day Time by PDFR Signalling to Dopaminergic Neurons in Drosophila melanogaster
Source: eNeuro. 2018 Aug 8;5(4):ENEURO.0129-18.2018. doi: 10.1523/ENEURO.0129-18.2018 (PMC6102377; doi:10.1523/ENEURO.0129-18.2018)
Supplement: Extended Data Figure 2-1 — One-way ANOVA with genotype as fixed factor conducted for day-time sleep of flies with downregulation of pdfr in indicated drivers. F(a-1), (N-k), where a is number of factor levels, N is the total number of replicates, and k refers to total number of groups. F statistic and p level of the main effect of genotype are indicated. Specific differences between genotypes determined after post hoc Tukey’s tests and indicated as asterisks in Figure 2A. Download Figure 2-1, DOCX file. [file sup_enu-eN-NWR-0129-18-s11.docx]

**Extended Data Figure 2-1**

|  | **Down-regulation of *pdfr*** | | |
| --- | --- | --- | --- |
| **Driver** | **F-statistic** | | ***p*** |
| ***Pdfr (B) GAL4*** | F_2,80_ | = 3.66 | < 0.05 |
| ***Cry-39 GAL4*** | F_2,86_ | = 5.89 | < 0.005 |
| ***Dvpdf GAL4*** | F_2,82_ | = 3.59 | < 0.05 |
| ***Pdf GAL4*** | F_2,91_ | = 6.18 | < 0 005 |
| ***Clk 9M GAL4*** | F_2,66_ | = 0.89 | 0.41 |
| ***Clk 4.1M GAL4*** | F_2,77_ | = 8.87 | < 0.0005 |
| ***Clk 4.5M GAL4*** | F_2,67_ | = 5.52 | < 0.05 |
|  |  |  |  |
| ***Dilp2 GAL4*** | F_2,54_ | = 2.47 | 0.09 |
| ***Kurs 45 GAL4*** | F_2,73_ | = 0.43 | =0.65 |
| ***Kurs 58 GAL4*** | F_2,79_ | = 2.9 | = 0.06 |
| ***Mai 281 GAL4*** | F_2,85_ | = 5.03 | < 0.05 |
| ***Mai 301 GAL4*** | F_2,82_ | = 12.38 | < 0.0005 |
|  |  |  |  |
| ***OK 107 GAL4*** | F_2,72_ | = 0.98 | = 0.38 |
| ***201y GAL4*** | F_2,88_ | = 2.02 | = 0.14 |
| ***c309 GAL4*** | F_2,87_ | = 8.89 | < 0.005 |
| ***c747 GAL4*** | F_2,75_ | = 4.43 | < 0.05 |
| ***30y GAL4*** | F_2,87_ | = 21.39 | < 0.00001 |
|  | | | |
| ***121y GAL4*** | F_2,80_ | = 13.24 | < 0.0005 |
| ***104y GAL4*** | F_2,69_ | = 3.87 | < 0.05 |
| ***c5 GAL4*** | F_2,69_ | = 6.37 | <0.005 |
| ***c119 GAL4*** | F_2,85_ | = 12.29 | < 0.0005 |
| ***c232 GAL4*** | F_2,89_ | = 6.69 | < 0.005 |
|  | | | |
| ***Ddc GAL4*** | F_2,90_ | = 4.87 | < 0.05 |
| ***TH GAL4*** | F_2,89_ | = 6.53 | < 0.005 |
| ***Tdc2 GAL4*** | F_2,64_ | = 4.3 | < 0.05 |
| ***Npf GAL4*** | F_2,91_ | 2.8 | = 0.07 |
