# Supplementary material for: Wakefulness Is Promoted during Day Time by PDFR Signalling to Dopaminergic Neurons in Drosophila melanogaster
Source: eNeuro. 2018 Aug 8;5(4):ENEURO.0129-18.2018. doi: 10.1523/ENEURO.0129-18.2018 (PMC6102377; doi:10.1523/ENEURO.0129-18.2018)
Supplement: Extended Data Figure 2-2 — One-way ANOVA with genotype as fixed factor conducted for day-time sleep of flies with OEX of pdfr in indicated drivers. F(a-1), (N-k), where a is number of factor levels, N is the total number of replicates and k refers to total number of groups. F statistic and p level of the main effect of genotype are indicated. Specific differences between genotypes determined after post hoc Tukey’s tests and indicated as asterisks in Figure 2B. Download Figure 2-2, DOCX file. [file sup_enu-eN-NWR-0129-18-s12.docx]

**Figure 2-2**

|  | **Over-expression of *pdfr*** | | |
| --- | --- | --- | --- |
| **Driver** | **F-statistic** | | ***p*** |
| ***Pdfr (B) GAL4*** | F_2,88_ | = 6.6 | < 0.005 |
| ***Cry-39 GAL4*** | F_2,86_ | = 5.2 | < 0.05 |
| ***Dvpdf GAL4*** | F_2,89_ | = 9.82 | < 0.0005 |
| ***Pdf GAL4*** | F_2,82_ | = 2.53 | 0.09 |
| ***Clk 9M GAL4*** | F_2,81_ | = 3.64 | < 0.05 |
| ***Clk 4.1M GAL4*** | F_2,78_ | = 1.6 | 0.2 |
| ***Clk 4.5M GAL4*** | F_2,85_ | = 1.6 | 0.2 |
|  | | | |
| ***Dilp2 GAL4*** | F_2,63_ | = 0.69 | 0.5 |
| ***Kurs 45 GAL4*** | F_2,93_ | = 34.93 | < 0.00001 |
| ***Kurs 58 GAL4*** | F_2,93_ | = 6.37 | < 0.005 |
| ***Mai 281 GAL4*** | F_2,91_ | = 13.46 | < 0.00001 |
| ***Mai 301 GAL4*** | F_2,89_ | = 11.44 | < 0.0005 |
|  | | | |
| ***OK 107 GAL4*** | F_2,80_ | = 0.49 | 0.61 |
| ***201y GAL4*** | F_2,88_ | = 1.55 | 0.22 |
| ***c309 GAL4*** | F_2,89_ | = 1.92 | 0.15 |
| ***c747 GAL4*** | F_2,81_ | = 1.06 | 0.35 |
| ***30y GAL4*** | F_2,89_ | = 4.01 | < 0.05 |
|  | | | |
| ***121y GAL4*** | F_2,86_ | = 98.63 | < 0.00001 |
| ***104y GAL4*** | F_2,89_ | = 27.48 | < 0.00001 |
| ***c5 GAL4*** | F_2,71_ | = 1.29 | 0.28 |
| ***c119 GAL4*** | F_2,88_ | = 2.58 | 0.08 |
| ***c232 GAL4*** | F_2,82_ | = 3.52 | < 0.04 |
|  | | | |
| ***Ddc GAL4*** | F_2,84_ | = 8.59 | < 0.0005 |
| ***TH GAL4*** | F_2,83_ | = 13.33 | < 0.0005 |
| ***Tdc2 GAL4*** | F_2,92_ | = 16.2 | < 0.00001 |
| ***Npf GAL4*** | F_2,83_ | = 0.6 | 0.55 |
