# Supplementary material for: Wakefulness Is Promoted during Day Time by PDFR Signalling to Dopaminergic Neurons in Drosophila melanogaster
Source: eNeuro. 2018 Aug 8;5(4):ENEURO.0129-18.2018. doi: 10.1523/ENEURO.0129-18.2018 (PMC6102377; doi:10.1523/ENEURO.0129-18.2018)
Supplement: Extended Data Figure 4-1 — One-way ANOVA with genotype as fixed factor conducted for day-time sleep of flies with downregulation of pdfr in indicated drivers. F(a-1), (N-k), where a is number of factor levels, N is the total number of replicates, and k refers to total number of groups. F statistic and p level of the main effect of genotype are indicated. Specific differences between genotypes determined after post hoc Tukey’s tests and indicated as asterisks in Figure 4A. Download Figure 4-1, DOCX file. [file sup_enu-eN-NWR-0129-18-s13.docx]

**Extended Data Figure 4-1**

|  | **Down-regulation of *pdfr*** | | |
| --- | --- | --- | --- |
| **Driver** | **F-statistic** | | ***p*** |
| ***TH-A* *GAL4*** | F_2,87_ | = 3.82 | < 0.05 |
| ***TH-C1 GAL4*** | F_2,89_ | = 0.13 | 0.87 |
| ***TH-C' GAL4*** | F_2,92_ | = 2.73 | 0.07 |
| ***TH-D1 GAL4*** | F_2,89_ | = 6.53 | < 0.005 |
| ***TH-D' GAL4*** | F_2,91_ | = 26.8 | < 0.00001 |
| ***TH-D4 GAL4*** | F_2,76_ | = 4.75 | < 0.05 |
| ***TH-F2 GAL4*** | F_2,91_ | = 0.57 | 0.57 |
| ***TH-F3 GAL4*** | F_2,72_ | = 14.08 | < 0.00001 |
| ***TH-G1 GAL4*** | F_2,88_ | = 11.66 | < 0.0005 |
