# Supplementary material for: Wakefulness Is Promoted during Day Time by PDFR Signalling to Dopaminergic Neurons in Drosophila melanogaster
Source: eNeuro. 2018 Aug 8;5(4):ENEURO.0129-18.2018. doi: 10.1523/ENEURO.0129-18.2018 (PMC6102377; doi:10.1523/ENEURO.0129-18.2018)
Supplement: Extended Data Figure 4-3 — Expression of GFP under TH GAL4, TH-D’, TH-D1, TH-D4, TH-F2, TH-F3, TH-G1 GAL4 drivers label different subsets of posterior protocerebrum lateral (PPL1-2) and medial (PPM1-3) neurons and their projections when posterior parts of the brains are imaged. Brains are costained with PDF for visualization of LNv and their projections. Scale bars: 20 µm. Download Figure 4-3, PDF file. [file sup_enu-eN-NWR-0129-18-s05.pdf]

Figure 4-3

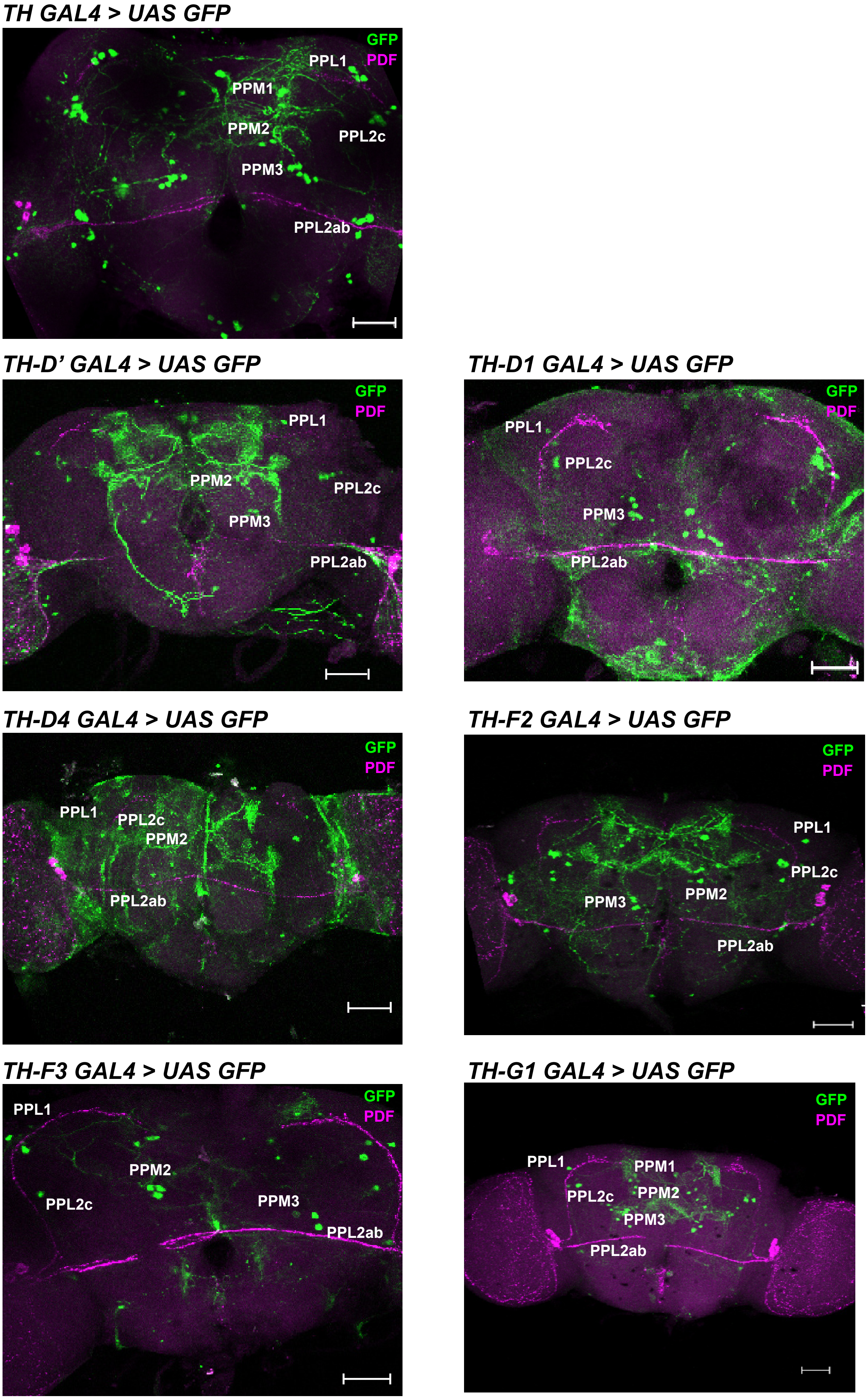

TH GAL4 > UAS GFP
GFP
PDF
PPL1
PPM1
PPM2
PPL2c
PPM3
PPL2ab
TH-D' GAL4 > UAS GFP
GFP
PDF
PPL1
PPM2
PPL2c
PPM3
PPL2ab
TH-D1 GAL4 > UAS GFP
GFP
PDF
PPL1
PPL2c
PPM3
PPL2ab
TH-D4 GAL4 > UAS GFP
GFP
PDF
PPL1
PPL2c
PPM2
PPL2ab
TH-F2 GAL4 > UAS GFP
GFP
PDF
PPL1
PPL2c
PPM3
PPM2
PPL2ab
TH-F3 GAL4 > UAS GFP
GFP
PDF
PPL1
PPM2
PPL2c
PPM3
PPL2ab
TH-G1 GAL4 > UAS GFP
GFP
PDF
PPL1
PPM1
PPL2c
PPM2
PPM3
PPL2ab
